# Macronutrient Optimization and Seasonal Diet Mixing in a Large Omnivore, the Grizzly Bear: A Geometric Analysis

**DOI:** 10.1371/journal.pone.0097968

**Published:** 2014-05-19

**Authors:** Sean C. P. Coogan, David Raubenheimer, Gordon B. Stenhouse, Scott E. Nielsen

**Affiliations:** 1 Department of Renewable Resources, University of Alberta, Edmonton, Alberta, Canada; 2 The Charles Perkins Centre, Faculty of Veterinary Science, and School of Biological Science, University of Sydney, Sydney, Australia; 3 Grizzly Bear Program, Foothills Research Institute, Hinton, AB, Canada; The University of Texas at San Antonio, United States of America

## Abstract

Nutrient balance is a strong determinant of animal fitness and demography. It is therefore important to understand how the compositions of available foods relate to required balance of nutrients and habitat suitability for animals in the wild. These relationships are, however, complex, particularly for omnivores that often need to compose balanced diets by combining their intake from diverse nutritionally complementary foods. Here we apply geometric models to understand how the nutritional compositions of foods available to an omnivorous member of the order Carnivora, the grizzly bear (*Ursus arctos* L.), relate to optimal macronutrient intake, and assess the seasonal nutritional constraints on the study population in west-central Alberta, Canada. The models examined the proportion of macronutrients that bears could consume by mixing their diet from food available in each season, and assessed the extent to which bears could consume the ratio of protein to non-protein energy previously demonstrated using captive bears to optimize mass gain. We found that non-selective feeding on ungulate carcasses provided a non-optimal macronutrient balance with surplus protein relative to fat and carbohydrate, reflecting adaptation to an omnivorous lifestyle, and that optimization through feeding selectively on different tissues of ungulate carcasses is unlikely. Bears were, however, able to dilute protein intake to an optimal ratio by mixing their otherwise high-protein diet with carbohydrate-rich fruit. Some individual food items were close to optimally balanced in protein to non-protein energy (e.g. *Hedysarum alpinum* roots), which may help explain their dietary prevalence. Ants may be consumed particularly as a source of lipids. Overall, our analysis showed that most food available to bears in the study area were high in protein relative to lipid or carbohydrate, suggesting the lack of non-protein energy limits the fitness (e.g. body size and reproduction) and population density of grizzly bears in this ecosystem.

## Introduction

A balanced macronutrient (protein, carbohydrate, and lipid) intake has been linked to many aspects of evolutionary fitness in animals, including fecundity [1], longevity [2], immune system function [3], obesity [4], sexual display [5], predation risk [6], body size and growth rate [7]–[8]. It may therefore be expected that animals are under strong selection for the ability to regulate the ratios of macronutrients eaten, through choosing foods that are balanced with respect to requirements or eating appropriate proportions of nutritionally complementary foods when consuming a mixed diet [9]. Laboratory studies have shown that a wide range of animals, including herbivores [7], [10], omnivores [11]–[12] and carnivores [9], [13]–[14] self-select specific ratios of macronutrients from nutritionally complementary foods, and that the selected ratios optimise performance [1], [7]–[8], [15]. Recent studies of primates, a group that is particularly amenable to direct observations of feeding in the field, have demonstrated that macronutrient ratios drive food selection also in the wild [16]–[18].

The generality, strength, and functional importance of macronutrient regulation provides a powerful framework for understanding the nutritional ecology of animals in the wild [19], and can inform management strategies for their conservation [20]. Few studies, however, have used the concepts of nutrient balance to understand the habitat requirements of animals, how they persist in natural environments in the face of temporal and spatial variation in food availability, and the nutritional constraints that limit populations. Such analyses are particularly pressing for large, vulnerable, ecologically important species that mix their diet from nutritionally diverse foods, such as omnivorous bears.

The natural diet of grizzly bears (*Ursus arctos* L.) has been well documented in parts of North America [21]–[25]. Being a generalist omnivore, the grizzly bear mixes its diet by consuming a variety of foods. Grizzly bear diets differ among regions, largely because of variation in food availability due to local climate and environmental factors [26], which results in individual and population level effects. For example, it has been suggested that the high fruit (carbohydrate) and low protein diet typical of grizzly bears in south-eastern British Columbia allows for a population with relatively small female bears with high body fat composition to live at high densities [27]. Much larger grizzly bears, and higher population densities, can be found in coastal areas where bears consume both fruit and salmon during the summer [28]–[29]. Food availability also varies temporally within grizzly bear populations, as bears consume food items that are seasonally available [21]–. Additionally, individual grizzly bear diets can be highly variable, ranging from near complete carnivory to almost totally plant-based [25], [30].

Macronutrient optimization has been documented in both captive and wild grizzly bears, which have been observed to self-select mixed diets of salmon (genus *Oncorhynchus*; a source of protein and lipid) and fruit (a source of carbohydrate) in proportions that optimized mass gain [31]. More recently, captive grizzly bears were shown to have optimized mass gain per unit energy intake on a self-selected average diet of 17% protein to 83% non-protein energy [32]. In the aforementioned study, grizzly bears prioritized a protein intake target over carbohydrate or lipid [32], which is common among terrestrial omnivores and herbivores [33]. Bears preferred lipid over carbohydrate as a non-protein energy source when optimizing protein intake. In the absence of lipid, however, bears were able to utilize carbohydrates with equal efficiency, suggesting that lipid and carbohydrate served as interchangeable non-protein-energy sources when optimizing protein intake [32].

In this paper, we used the geometric framework [8], [34] to understand how seasonal food availability may constrain macronutrient optimization in wild grizzly bears of west-central Alberta, Canada. Grizzly bears in the study area are relatively small, and occur at low population densities [35]. Since small body size and low population density (both indicators of performance) suggest poor environmental conditions, our analysis was aimed at understanding nutritional factors that may limit population fitness (e.g. body size and reproduction). To this end, we integrated previously published data on bear diets [32] with estimates of food nutritional composition in order to examine the proportion of macronutrients that grizzly bears could consume by mixing their diet among seasonally available foods. We then assessed the degree to which, in different seasons, bears could consume the ratio of protein to non-protein energy previously demonstrated to optimize mass gain in captive bears [32].

## Materials and Methods

### Study area and diet

West-central Alberta, Canada, (general location 53°0'N, 117°0'W) includes portions of the eastern slopes of the Canadian Rocky Mountains and foothills. The climate is continental, with average summer temperatures ranging from 11.9°C in lower elevation montane subregions, to 9.4°C in the subalpine, while average winter temperatures are −7.8°C and −8.9°C respectively [36]. Average annual rainfall in montane regions is 464 mm and 568 mm in the subalpine [36]. The grizzly bear population in the study area (4.79 bears per 1000 km^2^ [37]) resides in both mountainous and boreal foothills habitats, and large elevation gradients result in differences in resource distribution and timing of plant phenology that influence the dietary intake of bears [24], [38]–[39].

During the post-hibernation pre-green up period (approximately late April to late May) the diet of grizzly bears in the study area is composed primarily of alpine sweetvetch (*Hedysarum alpinum*) roots and ungulates, although small mammals can make up a portion of their diet [24].Ungulate consumption is higher throughout the active season among bears living in lower elevation foothills habitats than bears residing in mountainous areas, while sweetvetch roots are consumed more heavily and for longer periods by bears in the mountains. The principal ungulate prey of grizzly bear in the study area are moose (*Alces alces*), white-tailed deer (*Odocoileus virginianus*), mule deer (*O. hemionus*), and elk (*Cervus elaphus*) [24], [40]. Woodland caribou (*Rangifer tarandus caribou*) also occur in the more northerly Kakwa region of west-central Alberta. Ungulate consumption peaks in June and remains relatively high in July. Green vegetation becomes common in grizzly bear diets in June with consumption peaking in July. Commonly consumed native forbs include horsetails (*Equisetum* spp.) and cow parsnip (*Heracleum lanatum*) [24]. Forbs associated with anthropogenic disturbance (e.g., roads, oil and gas, mining, forestry, and seismic activity), such as dandelion (*Taraxacum officinale*), clover (*Trifolium* spp.), and alfalfa (*Medicago sattiva*) are also available to grizzly bears [24], [38], [40]. Sweetvetch root consumption is generally low during July; however, bears in the mountains continue to consume roots throughout the active season likely due to environmental gradients allowing for prolonged availability of nutritious roots and limited availability of animal-based foods [24], [39]. Fruit is prominent in the diet of grizzly bears in west-central Alberta from as early as late July, with consumption peaking in September [24]. Fruit most commonly consumed by bears in the study area include russet buffaloberry (*Shepherdia canadensis*) and those of the blueberry-huckleberry complex (*Vaccinium* spp.); however, several other species are consumed in lesser quantities [24]. The consumption of green vegetation declines during the fruit season, while ungulate consumption continues for bears in the foothills, but is lower among bears in the mountains [24]. Insects, primarily ants, are consumed mostly in July and August. In late fall, approximately mid-September to mid-October, a decline in the variety of available food resources has bears consuming a diet similar to the pre-green-up period: alpine sweetvetch roots once again becomes a primary food resource, and to a lesser extent ungulates [24]. Unlike the pregreen-up period, however, fruit is also often consumed by bears in the late-fall period [24].

### Macronutrient estimates

Macronutrient estimates for several food items were obtained from previous bear studies, the USDA National Nutrient Database [41], and other sources (Supporting Information S1). Chosen food items were considered generally sufficiently abundant for bears to effectively mix their diet based upon previous diet studies in the study area [24], [40]. We used available carbohydrate estimates for foods only when total dietary fibre (TDF) was reported in order to more closely approximate the digestion of bears [42] and to better compare with the study of Erlenbach et al. [32] where TDF was determined via the Prosky method. Fibre estimates, however, were not often reported as TDF, and we were therefore limited by a lack of suitable available carbohydrate estimates for foods, especially vegetation. Since complete or suitable macronutrient estimates for some vegetation were unknown to us, we included vegetation proxies and combined some estimates from different sources to estimate available carbohydrate (Supporting Information S1). For example, we used celery (*Apium graveolens* var. *dulce*) as a proxy for cow parsnip because they are similar in nutrient content [43]. Limited data analysis for some grizzly bear plant foods are presented in supplementary material (Supporting Information S1) and were used to inform macronutrient estimates. We used the USDA National Nutrient Database [41] to estimate macronutrient values for some wild ungulate tissues not reported in grizzly bear literature (including brain, kidney, liver, tongue, eyeball, and bone marrow) by using estimates from both wild and domestic animals (Supporting Information S1). We note that methods of diet composition may be different among studies, nutritional composition of food available to bears may differ from published data, and that food composition varies with respect to season and environment [21], [39], [44].

Since grizzly bears tend to consume ungulates in their entirety with the exception of hide and large bones [28], we estimated the macronutrient content of a wholly consumed ungulates (hereinafter referred to as “non-selective consumption”; Supporting Information S1). For the first estimate, we assumed an ungulate carcass was composed of five edible components (90.2% skeletal muscle, 1% brain, 3% bone marrow, 3.8% liver, and 2% adipose tissue) as in Kuipers et al. [45]. We then averaged macronutrient estimates, including available carbohydrate, for each ungulate component per season, weighted them according to the proportion of edible carcass, and summed the components to estimate the macronutrient content of a non-selectively consumed ungulate (Supporting Information S1). Estimates for bone marrow and adipose tissue were not averaged as we used only single estimates for each component. A second estimate of non-selective ungulate consumption for moose (minus hide and injesta) was derived from Hundertmark et al. [46] (Supporting Information S1). Carbohydrate was not reported in Hundertmark et al. [46], as such we assumed available carbohydrate to be negligible. A third model of non-selective ungulate consumption was derived from McCullough and Ullrey [47] for white-tailed deer. We used data presented in Table 1 and Table 2 of McCullough and Ullrey [47] to estimate the macronutrient composition of male and female deer of three different age classes (fawn, yearling and adult) minus hide (hair and skin), hooves, and antlers (where present). Estimates from McCullough and Ullrey [47] were derived from fall and winter animals following a high food resource year and as such were in excellent body condition (high body fat). We also determined the ratio of percent crude protein to lipid of an optimally balanced non-selectively consumed ungulate on a mass basis by back-calculating from the optimal ratio of 17% protein to 83% non-protein metabolizable energy (Supporting Information S1). We considered carbohydrates to be negligible for this estimate.

### Modelling approach

We used right-angled mixture triangles (RMTs) to examine the relationships between seasonal food availability to grizzly bears, macronutrient availability and macronutrient requirements [34] (see also [48]). RMTs represent 3-component (e.g. protein, fat and carbohydrate) compositions of mixtures as Cartesian points in a multi-dimensional nutrient space. We used Erlenbach et al. [32] as the basis for grizzly bear macronutrient requirements, where the ratio of 17% protein to 83% non-protein energy was considered optimal because it maximised growth efficiency. Grizzly bears also showed a strong regulatory preference for the protein intake target despite different diets, with lipid and carbohydrate being interchangeable components of non-protein energy intake [32]. Within RMTs fat and available carbohydrates were represented on the two primary (*x* and y) axes, and crude protein on the third, implicit, axis (the *z* axis). The implicit component varies inversely as distance from the origin increases [34]. Macronutrients were expressed as a percentage of total metabolizable energy (kcal) per food item (Supporting Information S1) using Atwater factors [49].

In order to examine temporal patterns in macronutrient availability, we created RMTs for four periods of the grizzly bear active season. These seasons were: 1) pre-green-up (approximately late April to late May); 2) forb and graminoid season (approximately June through July); 3) fruit season (approximately early August to mid-September); and 4) late fall (approximately mid-September to mid-October). After plotting food items, we created line segments between food points to form minimum convex polygons around the “nutrient space” potentially accessible to bears during each season. We used non-selective ungulate consumption estimates when creating nutrient space polygons in order to better approximate the nutrient space available to grizzly bears. The estimates from McCullough and Ullrey [47] were used for all seasons, but may overestimate the fat content of ungulates during pregreen-up, graminoid and forb, and berry seasons. Selective consumption of fatty ungulate components (e.g. bone, marrow and brain) were considered unlikely to contribute substantially to a balanced diet due to their size [45] and relative availability; however, we plotted macronutrient estimates of individual organs in order to illustrate the variety in nutrient composition among body parts. Small mammals were omitted from the nutrient space because they are generally a minor diet item and limited in size, although they may be a more prominent food in the diet of some bears ([24], unpublished data). In order to assess whether bears could achieve an optimal macronutrient intake, we plotted the intake target (17% protein to 83% non-protein energy) previously self-selected by captive bears, as well as the macronutrient composition of diets used to determine the intake target [32]. Overlap between the nutrient space polygon and intake target line would indicate that bears could optimize protein to non-protein energy intake by mixing their diet among seasonally available food.

## Results

### Pregreen-up period

**Figure 1:**
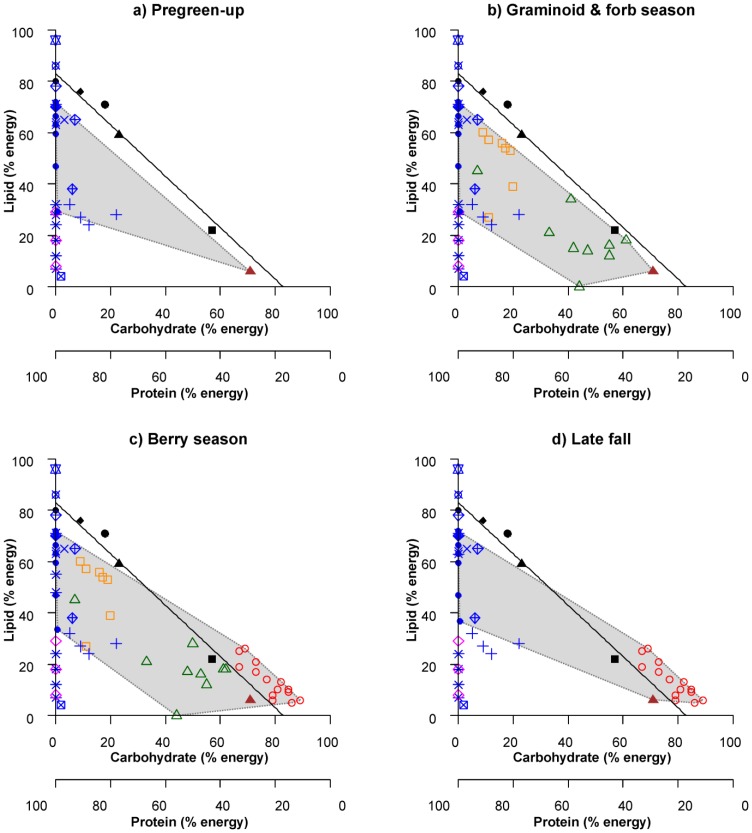
Right-angled mixture triangle (RMT) plots [34] depicting the estimated macronutrient (lipid, available carbohydrate, and crude protein) content of seasonally available foods consumed by grizzly bears in west-central Alberta, given as a percentage of metabolizable energy. Protein is represented by the third *z*-axis which varies inversely with distance from the origin. Seasons are defined based on major changes in grizzly bear diet, and include: a) pregreen-up; b) graminoid and forb season; c) berry season; and d) late fall. For reference, optimal diets self selected by captive grizzly bears [32] are shown as black symbols and marked in the legend with an asterisk (*), while the 17% protein to 83% non-protein energy intake target is shown as a black line. The grey-shaded polygon indicates the estimated nutrient space available to grizzly bears consuming seasonal foods. Overlap between the nutrient space polygon and the intake target line indicates that an optimal diet may be achieved during a season. The food items plotted do not include variation and are meant to give a general perspective.

RMT analysis of the macronutrient content of seasonal foods indicated that grizzly bears were unable to reach the macronutrient target of 17% protein during the pregreen-up period by mixing their diet between sweetvetch roots and whole ungulates (Fig. 1a); however, bears could come relatively close to the intake target by consuming sweetvetch root (23% protein: 77% non-protein energy), high-fat ungulates if available (28% protein: 72% non-protein energy), or a combination of the two. Alpine sweetvetch consumption would improve the macronutrient balance of bears consuming lower-fat ungulates such as our estimates for moose (53% protein: 47% non-protein energy) and average ungulate derived from Kuipers et al. [45] (70% protein: 30% non-protein energy) The carbohydrate content of brain and liver was estimated to contribute 0.5% to total metabolizable non-protein energy of an ungulate carcass based on the proportions of Kuipers et al [45]. In order for an ungulate carcass to be composed of an optimal ratio of protein to non-protein energy, it would need to have a percent mass ratio of crude protein to lipid of 0.433∶1 (Supporting Information S1). Conversely, an optimal ungulate carcass would need to contain approximately 2.3 X more lipid than protein on a mass basis. These ratios are the same for both percent dry matter and percent fresh (wet) matter estimates. Alpine sweetvetch root was close to optimally balanced in protein to non-protein energy due to its relatively high carbohydrate content. Small mammals would contribute mainly protein to the diet.

### Graminoid and forb season

During the graminoid and forb season, the addition of vegetation and ants to the diet allows bears to come closer to optimal protein intake by consuming a wider variety of carbohydrates and lipids (Fig. 1b). Specifically, a diet high in ants (approximately 30% protein: 70% lipid energy) would allow bears to compose a diet similar to the optimal lipid-rich diets chosen by captive bears, while some vegetation (horsetails and celery (proxy for cow parsnip)) had close to optimal ratios of macronutrients (25% protein: 75% non-protein energy, and 21% protein: 79% non-protein energy respectively). The ratio of protein to non-protein energy in available foods is higher than optimal during this season.

### Berry season

During the berry season, bears were able to optimize their macronutrient intake by mixing high-carbohydrate (67–89% metabolizable energy) fruit with all other food (ants, ungulates, sweetvetch root and green vegetation) in the nutrient space (Fig.1c). An optimal ratio of non-protein energy could be composed of a high carbohydrate/low lipid diet (approximately 78% carbohydrate and 5% lipid) to moderate carbohydrate/moderate lipid diet (approximately 33% carbohydrate and 50% lipid). Several individual fruits were close to optimally balanced in protein to non-protein energy.

### Late fall

During late fall, grizzly bears can still potentially optimize their protein to non-protein energy intake by consuming fruit with alpine sweetvetch and whole ungulates (Fig. 1d). In the absence of fruit, grizzly bears face a diet that is similar in macronutrient composition to the pregreen-up period.

## Discussion

Our analysis suggests that grizzly bears in west-central Alberta optimize macronutrient intake during seasons in which fruit is available. Fruit is commonly known as an important resource for bears, and is actively sought after, forming the bulk of bear diets during late-summer and early-fall when available [24], [50]. The ability of bears to optimize their macronutrient intake by consuming fruit helps explain why it is such a highly desirable food item. The timing of fruit availability is also critical: berry season coincides with the hyperphagic period in which bears attempt to accumulate sufficient fat reserves to support hibernation and female reproduction costs [51]–[53]. Beyond simply supplying energy to hyperphagic bears, fruit enables grizzly bears to optimize mass gain per unit energy intake. The availability of fruit, therefore, has direct implications on the reproductive success and fitness of grizzly bears in the study area.

Diet studies within the study area [24], [40] report that bears in the foothills have a much more carnivorous diet than bears in the mountains where ungulates and other animals are less abundant [24]. Our analysis shows that it is unlikely for grizzly bears to be able to optimize their diet by feeding non-selectively on ungulates, given that a balanced ungulate carcass would need to contain over two times the lipid as protein. For example, assuming an average crude protein content of 20.7% (fresh weight) for a moose carcass [46] would require a lipid content of 48.3% to be optimally balanced (assuming carbohydrates to be negligible). Such a high fat content is unlikely given that the average moose carcass in the same study contained 8.8% lipid with a range of 0.3–19.4% [46]. Additionally, the majority of ungulate consumption in the study area occurs during spring [24], a time when prey tend to be lean [44], [54]. However, despite not being perfectly balanced, ungulates provide more digestible protein and energy than plant-based grizzly bear foods [45]. Vegetation is complementary with ungulate consumption, in that it dilutes the level of protein in the diet and brings the diet closer to the protein intake target. This seems to be reflected in the diet of bears within the study area, which maintain the consumption of graminoids, forbs, and horsetails during periods of high ungulate intake [24]. Grizzly bears tend to gain lean mass in the spring [28] which seems consistent with a high protein diet.

Since fat-rich resources are relatively scarce in west-central Alberta, we would expect fatty ungulate tissues to be highly sought after. Selective feeding behaviour has been observed in Alaskan grizzly bears that fed on lipid-rich brain tissues and roe of spawning salmon [55]; however, as previously mentioned both wild and captive bears tend to consume ungulates in their entirety with the exception of hide and large bones [28]. It may be possible that bears in the study area selectively eat high fat ungulate tissues in order to increase their lipid intake, but presumably there are strong ecological constraints on availability to prevent bears from acquiring enough carcases to optimize their diet this way. This would be especially relevant for scavenged carcasses, as lipid rich tissue is often the first to be consumed and spoils more readily [45].

Certain individual foods were closely balanced in protein to non-protein energy, and these foods are also prominent in the grizzly bear diet. For example, celery (proxy for cow parsnip), horsetail, and dandelion were closely balanced in macronutrients. The near optimal macronutrient balance of sweetvetch root may help explain why it makes up much of the bear diet during pregreen-up and late fall [24]. Yet, despite favorable macronutrient balance, plant foods other than fruit are often low in nutrient concentration. Grizzly bears may respond to high levels of non-nutritional fibre and low macronutrient concentrations in roots, graminoids, and forbs by consuming these foods in greater quantities (a phenomenon observed in other animals [8]). Ultimately, however, grizzly bears are constrained by intake rates and stomach capacity when consuming energy- and macronutrient-dilute herbaceous foods [56]. Interestingly, for vegetation which we could estimate phenological changes in macronutrients, a decline in protein content from graminoid and forb season to berry season made these plants more closely balanced in macronutrients; however, nutrient concentration in these plants is generally lower and fibre content higher [21], [23].

The macronutrient balance of ants makes them a potentially important food item for grizzly bears. Our results suggest that lipid content of ants makes them a particularly valuable food resource, because they allow bears to expand their nutrient space in the absence of high-fat ungulates and consume a diet that is close to the balance offered by preferred high-lipid diets. As well, ants have previously been suggested to provide necessary amino acids to bears on largely vegetarian diets [57]. Ants, however, are generally not heavily consumed by grizzly bears in the study area and only for a limited time [24]. Therefore, the dietary impact of ants on the study area population is likely limited.

Most food available to bears in the study area were high in protein relative to lipid or carbohydrate, and it is not unreasonable to suggest that a paucity of lipid-rich resources in part limits the study area population in terms of body size and population density relative to bears in ecosystems that provide more fatty foods (e.g., Yellowstone, and coastal British Columbia/Alaska). Yet, in the face of a suboptimal diet, generalist omnivores such as the grizzly bear may have a high capacity for capitalizing on excess nutrients in nutritionally imbalanced foods, even if it means deviating further from optimal nutrient intake [8]. One explanation for this is that a generalist species that has over-ingested a particular macronutrient has a relatively higher probability of encountering a complimentary food that enables it to correct the nutrient imbalance, thereby rendering the initial excess nutrient useable while also balancing the nutrient deficiency in the complimentary food [58]. This ability to capitalize on excess nutrients may be especially true of animals that readily store excess energy as fat [58], which likely includes grizzly bears. Given that grizzly bears tend to consume large amounts of seasonal food resources, they may be able to effectively mix their diet across longer time periods, which has been previously suggested [27], but has yet to be demonstrated.

While an optimal ratio of protein to non-protein energy could be consumed by grizzly bears in west-central Alberta, the absolute amount of food consumed would need to be adequate to support maintenance and growth. The possibility for growth is obviously not always available to bears given inter-annual variation in both ungulate densities and berry production. Other factors may limit the availability of food resources to individual grizzly bears, including sexual segregation of habitats [30]. Yet despite the challenges, years of high resource availability should be those in which bears experience the greatest growth, not solely because of increased energy availability, but also due to the increased potential for optimizing macronutrient intake through a mixed diet.

As with other wild animals [59]–[60], there are knowledge gaps regarding the nutritional compositions of resources consumed by grizzly bears, and, as in many nutritional ecology studies [60], protein and energy dominate the nutritional currencies used when investigating grizzly bear ecology. While such an approach is often appropriate, this work further demonstrates the importance of expanding the study of grizzly bear nutritional ecology to include other nutrients and the interactions between them. Indeed, an important priority is to extend the dimensionality of such models, to include not only macronutrients but also, for example, essential amino and fatty acids as well as micronutrients. Such studies are, of course, challenging, but we believe that the power of multi-dimensional nutritional analyses outweighs the effort [33].

## Supporting Information

Supporting Information S1 Nutritional estimates and information used to model the macronutrient content of seasonal grizzly bear foods in west-central Alberta, Canada.
(DOC)
