# Correction: Macronutrient Optimization and Seasonal Diet Mixing in a Large Omnivore, the Grizzly Bear: A Geometric Analysis

**DOI:** 10.1371/journal.pone.0105719

**Published:** 2014-08-12

**Authors:** The PLOS ONE Staff

Figure 1 is missing the explanatory legend. The correct version of Figure 1 alongside the key can be viewed below.

**Figure 1:**
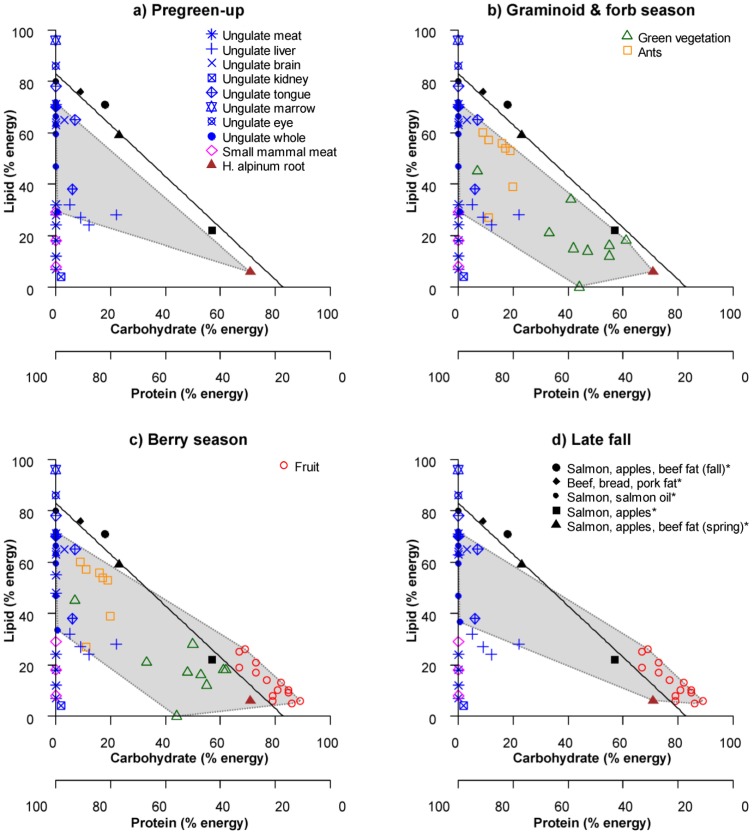
Right-angled mixture triangle (RMT) plots [34] depicting the estimated macronutrient (lipid, available carbohydrate, and crude protein) content of seasonally available foods consumed by grizzly bears in west-central Alberta, given as a percentage of metabolizable energy. Protein is represented by the third z-axis which varies inversely with distance from the origin. Seasons are defined based on major changes in grizzly bear diet, and include: a) pregreen-up; b) graminoid and forb season; c) berry season; and d) late fall. For reference, optimal diets self selected by captive grizzly bears [32] are shown as black symbols and marked in the legend with an asterisk (*), while the 17% protein to 83% non-protein energy intake target is shown as a black line. The grey-shaded polygon indicates the estimated nutrient space available to grizzly bears consuming seasonal foods. Overlap between the nutrient space polygon and the intake target line indicates that an optimal diet may be achieved during a season. The food items plotted do not include variation and are meant to give a general perspective.
