# Supplementary material for: Macronutrient Optimization and Seasonal Diet Mixing in a Large Omnivore, the Grizzly Bear: A Geometric Analysis
Source: PLoS One. 2014 May 19;9(5):e97968. doi: 10.1371/journal.pone.0097968 (PMC4026535; doi:10.1371/journal.pone.0097968)
Supplement: Supporting Information S1 — Nutritional estimates and information used to model the macronutrient content of seasonal grizzly bear foods in west-central Alberta, Canada. (DOC) [file pone.0097968.s001.doc]

Supporting Information for the article

“Macronutrient Optimization and Seasonal Diet Mixing in a Large Omnivore, the Grizzly Bear: a Geometric Analysis”

Sean C. P. Coogan^1^*, David Raubenheimer^2^, G. B. Stenhouse^3^, Scott E. Nielsen^1^

*(1) Department of Renewable Resources, University of Alberta, Edmonton, Alberta, Canada*

*(2) The Charles Perkins Centre, Faculty of Veterinary Science, and School of Biological Science, University of Sydney, Australia*

*(3) Grizzly Bear Program, Foothills Research Institute, 1176 Switzer Drive, Hinton, AB, Canada*

* Corresponding author: Sean C. P. Coogan. Email: scoogan@ualberta.ca; sean.coogan@sydney.edu.au

Corresponding author current affiliation: School of Biological Sciences and the Charles Perkins Centre, University of Sydney, Australia

**Supporting Information S1**

Table 1 Food groups, diet items, percent (dry matter) macronutrient (crude protein, available carbohydrate, and lipid) composition, metabolic (Atwater) conversion factors (kcal/g; Merrill and Watt 1973), and source of nutrient estimates used to model seasonal macronutrient availability for grizzly bears in west-central Alberta, Canada.

| Food Group | Diet Items | Protein | Carbohydrate | Lipid |  | Protein | Carbohydrate | Lipid |  | Source |
| --- | --- | --- | --- | --- | --- | --- | --- | --- | --- | --- |
|  |  | (% dry matter) | | |  | (metabolic conversion factors (kcal/g)) | | |  |  |
| ***Pre-green up*** |  |  |  |  |  |  |  |  |  |  |
| *H. alpinum* -- root | *Hedysarum alpinum* root§ | 12.6 | 33.8 | 1.5 |  | 3.47 | 4.07 | 8.37 |  | Coogan 2012 |
| Ungulate -- meat | Moose | 81.0 | 0 | 15.0 |  | 4.27 | 0 | 9.02 |  | Mattson et al. 2004 |
| Ungulate -- meat | Moose meat, raw | 90.1 | 0 | 3.0 |  | 4.27 | 0 | 9.02 |  | USDA 2013 |
| Ungulate -- meat | Moose meat, raw, Alaska | 88.5 | 0 | 5.6 |  | 4.27 | 0 | 9.02 |  | USDA 2013 |
| Ungulate -- meat | Deer | 45.1 | 0 | 52.9 |  | 4.27 | 0 | 9.02 |  | Pritchard and Robbins 1990 |
| Ungulate -- meat | Deer, raw, game meat | 86.9 | 0 | 9.2 |  | 4.27 | 0 | 9.02 |  | USDA 2013 |
| Ungulate -- meat | Elk | 80.0 | 0 | 18.0 |  | 4.27 | 0 | 9.02 |  | Mattson et al. 2004 |
| Ungulate -- meat | Elk, game meat, raw | 89.6 | 0 | 5.7 |  | 4.27 | 0 | 9.02 |  | USDA 2013 |
| Ungulate -- meat | Caribou, meat, raw† | 79.3 | 0 | 11.8 |  | 4.27 | 0 | 9.02 |  | USDA 2013 |
| Ungulate -- meat | Caribou, hind quarter, meat, raw, Alaska† | 82.6 | 0 | 12.3 |  | 4.27 | 0 | 9.02 |  | USDA 2013 |
| Ungulate -- liver | Caribou, liver, raw, Alaska† | 52.4 | 23.8 | 13.6 |  | 4.27 | 4.11 | 9.02 |  | USDA 2013 |
| Ungulate -- liver | Moose, liver, braised, Alaska | 71.7 | 10.1 | 14.4 |  | 4.27 | 4.11 | 9.02 |  | USDA 2013 |
| Ungulate -- liver | Lamb, liver, raw* | 71.2 | 6.2 | 17.5 |  | 4.27 | 4.11 | 9.02 |  | USDA 2013 |
| Ungulate -- liver | Beef, liver, raw* | 69.7 | 13.3 | 12.4 |  | 4.27 | 4.11 | 9.02 |  | USDA 2013 |
| Ungulate -- brain | Beef, brain, raw* | 45.8 | 4.4 | 43.4 |  | 4.27 | 4.11 | 9.02 |  | USDA 2013 |
| Ungulate -- brain | Lamb, brain, raw* | 50.0 | 0 | 41.3 |  | 4.27 | 4.11 | 9.02 |  | USDA 2013 |
| Ungulate -- brain | Veal, brain, raw* | 51.0 | 0 | 40.6 |  | 4.27 | 4.11 | 9.02 |  | USDA 2013 |
| Ungulate -- kidney | Beef, kidneys, raw* | 78.7 | 1.3 | 1.5 |  | 4.27 | 4.11 | 9.02 |  | USDA 2013 |
| Ungulate -- tongue | Caribou, tongue, raw, Alaska† | 36.5 | 0 | 60.8 |  | 4.27 | 4.11 | 9.02 |  | USDA 2013 |
| Ungulate -- tongue | Beef, tongue, raw* | 42.0 | 10.4 | 45.7 |  | 4.27 | 4.11 | 9.02 |  | USDA 2013 |
| Ungulate -- tongue | Lamb, tongue, raw* | 46.2 | 0 | 52.1 |  | 4.27 | 4.11 | 9.02 |  | USDA 2013 |
| Ungulate -- tongue | Veal, tongue, raw* | 67.5 | 7.5 | 21.5 |  | 4.27 | 4.11 | 9.02 |  | USDA 2013 |
| Ungulate -- marrow | Caribou, bone marrow, raw† | 8.0 | 0 | 91.1 |  | 4.27 | 0 | 9.02 |  | USDA 2013 |
| Ungulate -- eye | Caribou,eye, raw, Alaska† | 25.2 | 0 | 73.2 |  | 4.27 | 0 | 9.02 |  | USDA 2013 |
| Small mammal -- meat | Squirrel, raw, game meat | 81.7 | 0 | 3.2 |  | 4.27 | 0 | 9.02 |  | USDA 2013 |
| Small mammal -- meat | Squirrel, ground, meat, Alaska | 78.5 | 0 | 15.4 |  | 4.27 | 0 | 9.02 |  | USDA 2013 |
| Small mammal -- meat | Rabbit, wild, raw, game meat | 85.5 | 0 | 9.1 |  | 4.27 | 0 | 9.02 |  | USDA 2013 |
| ***Graminoid & forb season*** | |  |  |  |  |  |  |  |  |  |
| Green vegetation | Celery‡ | 15.1 | 30 | 3.7 |  | 2.44 | 3.57 | 8.37 |  | USDA 2013 |
| Green vegetation | Clover | 27.6 | 18.1 | 2.7 |  | 2.44 | 3.57 | 8.37 |  | Rode et al 2001; Tran 2013 |
| Green vegetation | Graminoids | 20.5 | 16.9 | 2.2 |  | 2.44 | 3.57 | 8.37 |  | Rode et al. 2001; Hueze et al 2011 |
| Green vegetation | Horsetails | 14 | 15.3 | 5.4 |  | 2.44 | 3.57 | 8.37 |  | Thomas and Prevett 1982; Coogan 2012 |
| Green vegetation | Dandelion | 30 | 39.6 | 4.86 |  | 2.44 | 3.57 | 8.37 |  | USDA 2013; Online Resource (this paper) |
| Green vegetation | Fireweed, young leaves, raw, Alaska± | 23.4 | 2.3 | 6.3 |  | 2.44 | 3.57 | 8.37 |  | USDA 2013 |
| Green vegetation | Spinach** | 33.3 | 16.6 | 4.5 |  | 2.44 | 3.57 | 8.37 |  | USDA 2013 |
| Green vegetation | Lettuce, green leaf, raw** | 27.1 | 31.3 | 3 |  | 2.44 | 3.57 | 8.37 |  | USDA 2013 |
| Green vegetation | Cattail, Narrow Leaf, shoots, Typha lattifolia± | 16.1 | 8.7 | 0 |  | 2.44 | 3.57 | 8.37 |  | USDA 2013 |
| Insects -- ants | Ant - Formica spp. Worker | 27 | 20.3 | 24 |  | 4.27 | 3.87 | 9.02 |  | Noyce et al. 1997 |
| Insects -- ants | Ant - Acanthomyops spp. workers 1 | 37.6 | 11.9 | 33.9 |  | 4.27 | 3.87 | 9.02 |  | Noyce et al. 1997 |
| Insects -- ants | Ant - Acanthomyops spp. workers 2 | 32 | 20.4 | 28.2 |  | 4.27 | 3.87 | 9.02 |  | Noyce et al. 1997 |
| Insects -- ants | Ant - Acanthomyops spp. pupae 1 | 43.9 | 15.6 | 35.7 |  | 4.27 | 3.87 | 9.02 |  | Noyce et al. 1997 |
| Insects -- ants | Ant - Acanthomyops spp. pupae 2 | 36.3 | 22.4 | 33.5 |  | 4.27 | 3.87 | 9.02 |  | Noyce et al. 1997 |
| Insects -- ants | Ant & pupae - *Camponotus herculeanus* | 43.8 | 23.5 | 19.4 |  | 4.27 | 3.87 | 9.02 |  | Swenson et al. 1999 |
| Insects -- ants | Ant & pupae - *Formica* spp. | 55.9 | 10.4 | 11.3 |  | 4.27 | 3.87 | 9.02 |  | Swenson et al. 1999 |
| Ungulate -- meat | Moose | 81 | 0 | 15 |  | 4.27 | 0 | 9.02 |  | Mattson et al. 2004 |
| Ungulate -- meat | Moose meat, raw | 90.1 | 0 | 3 |  | 4.27 | 0 | 9.02 |  | USDA 2013 |
| Ungulate -- meat | Moose meat, raw, Alaska | 88.5 | 0 | 5.6 |  | 4.27 | 0 | 9.02 |  | USDA 2013 |
| Ungulate -- meat | Deer | 45.1 | 0 | 52.9 |  | 4.27 | 0 | 9.02 |  | Pritchard and Robbins 1990 |
| Ungulate -- meat | Deer, raw, game meat | 86.9 | 0 | 9.2 |  | 4.27 | 0 | 9.02 |  | USDA 2013 |
| Ungulate -- meat | Elk | 80 | 0 | 18 |  | 4.27 | 0 | 9.02 |  | Mattson et al. 2004 |
| Ungulate -- meat | Elk, game meat, raw | 89.6 | 0 | 5.7 |  | 4.27 | 0 | 9.02 |  | USDA 2013 |
| Ungulate -- meat | Caribou, meat, raw† | 79.3 | 0 | 11.8 |  | 4.27 | 0 | 9.02 |  | USDA 2013 |
| Ungulate -- meat | Caribou, hind quarter, meat, raw, Alaska† | 82.6 | 0 | 12.3 |  | 4.27 | 0 | 9.02 |  | USDA 2013 |
| Ungulate -- liver | Caribou, liver, raw, Alaska† | 52.4 | 23.8 | 13.6 |  | 4.27 | 4.11 | 9.02 |  | USDA 2013 |
| Ungulate -- liver | Moose, liver, braised, Alaska | 71.7 | 10.1 | 14.4 |  | 4.27 | 4.11 | 9.02 |  | USDA 2013 |
| Ungulate -- liver | Lamb, liver, raw | 71.2 | 6.2 | 17.5 |  | 4.27 | 4.11 | 9.02 |  | USDA 2013 |
| Ungulate -- liver | Beef, liver, raw* | 69.7 | 13.3 | 12.4 |  | 4.27 | 4.11 | 9.02 |  | USDA 2013 |
| Ungulate -- brain | Beef, brain, raw* | 45.8 | 4.4 | 43.4 |  | 4.27 | 4.11 | 9.02 |  | USDA 2013 |
| Ungulate -- brain | Lamb, brain, raw* | 50 | 0 | 41.25 |  | 4.27 | 4.11 | 9.02 |  | USDA 2013 |
| Ungulate -- brain | Veal, brain, raw* | 51 | 0 | 40.6 |  | 4.27 | 4.11 | 9.02 |  | USDA 2013 |
| Ungulate -- kidney | Beef, kidneys, raw* | 78.7 | 1.3 | 1.5 |  | 4.27 | 4.11 | 9.02 |  | USDA 2013 |
| Ungulate -- tongue | Caribou, tongue, raw, Alaska† | 36.5 | 0 | 60.8 |  | 4.27 | 4.11 | 9.02 |  | USDA 2013 |
| Ungulate -- tongue | Beef, tongue, raw* | 42 | 10.4 | 45.7 |  | 4.27 | 4.11 | 9.02 |  | USDA 2013 |
| Ungulate -- tongue | Lamb, tongue, raw* | 46.2 | 0 | 52.1 |  | 4.27 | 4.11 | 9.02 |  | USDA 2013 |
| Ungulate -- tongue | Veal, tongue, raw* | 67.5 | 7.5 | 21.5 |  | 4.27 | 4.11 | 9.02 |  | USDA 2013 |
| Ungulate -- marrow | Caribou, bone marrow, raw† | 8 | 0 | 91.1 |  | 4.27 | 0 | 9.02 |  | USDA 2013 |
| Ungulate -- eye | Caribou,eye, raw, Alaska† | 25.2 | 0 | 73.2 |  | 4.27 | 0 | 9.02 |  | USDA 2013 |
| Small mammal -- meat | Squirrel, raw, game meat | 81.7 | 0 | 3.21 |  | 4.27 | 0 | 9.02 |  | USDA 2013 |
| Small mammal -- meat | Squirrel, ground, meat, Alaska | 78.5 | 0 | 15.4 |  | 4.27 | 0 | 9.02 |  | USDA 2013 |
| Small mammal -- meat | Rabbit, wild, raw, game meat | 85.5 | 0 | 9.1 |  | 4.27 | 0 | 9.02 |  | USDA 2013 |
| H. alpinum -- root | *Hedysarum alpinum* root§ | 12.6 | 33.8 | 1.5 |  | 3.47 | 4.07 | 8.37 |  | Coogan 2012 |
| ***Berry season*** | |  |  |  |  |  |  |  |  |  |
| Fruit | *Vaccinium myrtilloides* | 4.7 | 64.9 | 2.9 |  | 3.36 | 3.6 | 8.37 |  | Coogan 2012 |
| Fruit | *Vaccinium vits-idaea* | 4.4 | 66.6 | 3.3 |  | 3.36 | 3.6 | 8.37 |  | Coogan 2012 |
| Fruit | *Vaccinum scoparium* | 4.9 | 65.4 | 4.4 |  | 3.36 | 3.6 | 8.37 |  | Coogan 2012 |
| Fruit | Buffaloberry (*Shepherdia canadensis*) | 3.8 | 50.7 | 8.3 |  | 3.36 | 3.6 | 8.37 |  | Coogan 2012; Online Resource (this paper) |
| Fruit | Crowberry (*Empetrum nigrum*) | 3.5 | 42.4 | 5.3 |  | 3.36 | 3.6 | 8.37 |  | Coogan 2012 |
| Fruit | Raspberry, raw | 8.4 | 38.1 | 4.6 |  | 3.36 | 3.6 | 8.37 |  | USDA 2013 |
| Fruit | Raspberries, wild | 7.2 | 40.9 | 1.8 |  | 3.36 | 3.6 | 8.37 |  | USDA 2013 |
| Fruit | Strawberries, raw | 7.4 | 62.8 | 3.3 |  | 3.36 | 3.6 | 8.37 |  | USDA 2013 |
| Fruit | Gooseberries, raw, *Ribes* spp. | 7.3 | 48.5 | 4.8 |  | 3.36 | 3.6 | 8.37 |  | USDA 2013 |
| Fruit | Cranberries, wild, bush, raw, Alaska, *Vibernum edule* | 7.9 | 40 | 1.4 |  | 3.36 | 3.6 | 8.37 |  | USDA 2013 |
| Fruit | Blackberries, wild, raw, Alaska, crowberry, Rubus spp. | 7.03 | 55.6 | 9 |  | 3.36 | 3.6 | 8.37 |  | USDA 2013 |
| Fruit | Blueberries, wild, raw, Alaska, *Vaccinium ovalifolium#* | 8.4 | 66.9 | 5.2 |  | 3.36 | 3.6 | 8.37 |  | USDA 2013t |
| Fruit | Blueberries, raw | 4.7 | 76.6 | 2.1 |  | 3.36 | 3.6 | 8.37 |  | USDA 2013 |
| Fruit | Rose Hips, wild | 3.9 | 34.2 | 0.8 |  | 3.36 | 3.6 | 8.37 |  | USDA 2013 |
| Green vegetation | Celery‡ | 15.1 | 30 | 3.7 |  | 2.44 | 3.57 | 8.37 |  | USDA 2013 |
| Green vegetation | Clover | 19.5 | 18 | 2.7 |  | 2.44 | 3.57 | 8.37 |  | Rode et al 2001; Tran 2013 |
| Green vegetation | Graminoids | 15 | 17.7 | 2.2 |  | 2.44 | 3.57 | 8.37 |  | Rode et al 2001; Mattson et al. 2004; Hueze et al 2011 |
| Green vegetation | Horsetails | 11.2 | 17.5 | 4.1 |  | 2.44 | 3.57 | 8.37 |  | Thomas and Prevett 1982; Coogan 2012 |
| Green vegetation | Dandelion | 18.8 | 39.6 | 4.9 |  | 2.44 | 3.57 | 8.37 |  | USDA 2013 |
| Green vegetation | Fireweed, young leaves, raw, Alaska± | 23.4 | 2.3 | 6.3 |  | 2.44 | 3.57 | 8.37 |  | USDA 2013 |
| Green vegetation | Spinach** | 33.3 | 16.6 | 4.5 |  | 2.44 | 3.57 | 8.37 |  | USDA 2013 |
| Green vegetation | Lettuce, green leaf, raw** | 27.1 | 31.3 | 3 |  | 2.44 | 3.57 | 8.37 |  | USDA 2013 |
| Green vegetation | Cattail, Narrow Leaf, shoots, Typha lattifolia± | 16.1 | 8.7 | 0 |  | 2.44 | 3.57 | 8.37 |  | USDA 2013 |
| Insects -- ants | Ant - Formica spp. Worker | 27 | 20.3 | 24 |  | 4.27 | 3.87 | 9.02 |  | Noyce et al. 1997 |
| Insects -- ants | Ant - Acanthomyops spp. workers 1 | 37.6 | 11.9 | 33.9 |  | 4.27 | 3.87 | 9.02 |  | Noyce et al. 1997 |
| Insects -- ants | Ant - Acanthomyops spp. workers 2 | 32 | 20.4 | 28.2 |  | 4.27 | 3.87 | 9.02 |  | Noyce et al. 1997 |
| Insects -- ants | Ant - Acanthomyops spp. pupae 1 | 43.9 | 15.6 | 35.7 |  | 4.27 | 3.87 | 9.02 |  | Noyce et al. 1997 |
| Insects -- ants | Ant - Acanthomyops spp. pupae 2 | 36.3 | 22.4 | 33.5 |  | 4.27 | 3.87 | 9.02 |  | Noyce et al. 1997 |
| Insects -- ants | Ant & pupae - Camponotus herculeanus | 43.8 | 23.5 | 19.4 |  | 4.27 | 3.87 | 9.02 |  | Swenson et al. 1999 |
| Insects -- ants | Ant & pupae - Formica spp. | 55.9 | 10.4 | 11.3 |  | 4.27 | 3.87 | 9.02 |  | Swenson et al. 1999 |
| Ungulate -- meat | Moose | 67 | 0 | 29 |  | 4.27 | 0 | 9.02 |  | Mattson et al. 2004 |
| Ungulate -- meat | Moose meat, raw | 90.1 | 0 | 3 |  | 4.27 | 0 | 9.02 |  | USDA 2013 |
| Ungulate -- meat | Moose meat, raw, Alaska | 88.5 | 0 | 5.6 |  | 4.27 | 0 | 9.02 |  | USDA 2013 |
| Ungulate -- meat | Deer | 45.1 | 0 | 52.9 |  | 4.27 | 0 | 9.02 |  | Pritchard and Robbins 1990 |
| Ungulate -- meat | Deer, raw, game meat | 86.9 | 0 | 9.2 |  | 4.27 | 0 | 9.02 |  | USDA 2013 |
| Ungulate -- meat | Elk | 62 | 0 | 36 |  | 4.27 | 0 | 9.02 |  | Mattson et al. 2004 |
| Ungulate -- meat | Elk, game meat, raw | 89.6 | 0 | 5.7 |  | 4.27 | 0 | 9.02 |  | USDA 2013 |
| Ungulate -- meat | Caribou, meat, raw† | 79.3 | 0 | 11.8 |  | 4.27 | 0 | 9.02 |  | USDA 2013 |
| Ungulate -- meat | Caribou, hind quarter, meat, raw, Alaska† | 82.6 | 0 | 12.3 |  | 4.27 | 0 | 9.02 |  | USDA 2013 |
| Ungulate -- liver | Caribou, liver, raw, Alaska† | 52.4 | 23.8 | 13.6 |  | 4.27 | 4.11 | 9.02 |  | USDA 2013 |
| Ungulate -- liver | Moose, liver, braised, Alaska | 71.7 | 10.1 | 14.4 |  | 4.27 | 4.11 | 9.02 |  | USDA 2013 |
| Ungulate -- liver | Lamb, liver, raw* | 71.2 | 6.2 | 17.5 |  | 4.27 | 4.11 | 9.02 |  | USDA 2013 |
| Ungulate -- liver | Beef, liver, raw* | 69.7 | 13.3 | 12.4 |  | 4.27 | 4.11 | 9.02 |  | USDA 2013 |
| Ungulate -- brain | Beef, brain, raw* | 45.8 | 4.4 | 43.4 |  | 4.27 | 4.11 | 9.02 |  | USDA 2013 |
| Ungulate -- brain | Lamb, brain, raw* | 50 | 0 | 41.25 |  | 4.27 | 4.11 | 9.02 |  | USDA 2013 |
| Ungulate -- brain | Veal, brain, raw* | 51 | 0 | 40.6 |  | 4.27 | 4.11 | 9.02 |  | USDA 2013 |
| Ungulate -- kidney | Beef, kidneys, raw* | 78.7 | 1.3 | 1.5 |  | 4.27 | 4.11 | 9.02 |  | USDA 2013 |
| Ungulate -- tongue | Caribou, tongue, raw, Alaska† | 36.5 | 0 | 60.8 |  | 4.27 | 4.11 | 9.02 |  | USDA 2013 |
| Ungulate -- tongue | Beef, tongue, raw* | 42 | 10.4 | 45.7 |  | 4.27 | 4.11 | 9.02 |  | USDA 2013 |
| Ungulate -- tongue | Lamb, tongue, raw* | 46.2 | 0 | 52.1 |  | 4.27 | 4.11 | 9.02 |  | USDA 2013 |
| Ungulate -- tongue | Veal, tongue, raw* | 67.5 | 7.5 | 21.5 |  | 4.27 | 4.11 | 9.02 |  | USDA 2013 |
| Ungulate -- marrow | Caribou, bone marrow, raw† | 8 | 0 | 91.1 |  | 4.27 | 0 | 9.02 |  | USDA 2013 |
| Ungulate -- eye | Caribou,eye, raw, Alaska† | 25.2 | 0 | 73.2 |  | 4.27 | 0 | 9.02 |  | USDA 2013 |
| Small mammal -- meat | Squirrel, raw, game meat | 81.7 | 0 | 3.21 |  | 4.27 | 0 | 9.02 |  | USDA 2013 |
| Small mammal -- meat | Squirrel, ground, meat, Alaska | 78.5 | 0 | 15.4 |  | 4.27 | 0 | 9.02 |  | USDA 2013 |
| Small mammal -- meat | Rabbit, wild, raw, game meat | 85.5 | 0 | 9.1 |  | 4.27 | 0 | 9.02 |  | USDA 2013 |
| H. alpinum -- root | *Hedysarum alpinum* root§ | 12.6 | 33.8 | 1.5 |  | 3.47 | 4.07 | 8.37 |  | Coogan 2012 |
| ***Late fall*** | |  |  |  |  |  |  |  |  |  |
| Fruit | *Vaccinium myrtilloides* | 4.7 | 64.9 | 2.9 |  | 3.36 | 3.6 | 8.37 |  | Coogan 2012 |
| Fruit | *Vaccinium vits-idaea* | 4.4 | 66.6 | 3.3 |  | 3.36 | 3.6 | 8.37 |  | Coogan 2012 |
| Fruit | *Vaccinum scoparium* | 4.9 | 65.4 | 4.4 |  | 3.36 | 3.6 | 8.37 |  | Coogan 2012 |
| Fruit | Buffaloberry (*Shepherdia canadensis*) | 3.8 | 50.7 | 8.3 |  | 3.36 | 3.6 | 8.37 |  | Coogan 2012; Online Resource (this paper) |
| Fruit | Crowberry (*Empetrum nigrum*) | 3.5 | 42.4 | 5.3 |  | 3.36 | 3.6 | 8.37 |  | Coogan 2012 |
| Fruit | Raspberry, raw | 8.4 | 38.1 | 4.6 |  | 3.36 | 3.6 | 8.37 |  | USDA 2013 |
| Fruit | Raspberries, wild | 7.2 | 40.9 | 1.8 |  | 3.36 | 3.6 | 8.37 |  | USDA 2013 |
| Fruit | Strawberries, raw | 7.4 | 62.8 | 3.3 |  | 3.36 | 3.6 | 8.37 |  | USDA 2013 |
| Fruit | Gooseberries, raw, Ribes spp. | 7.3 | 48.5 | 4.8 |  | 3.36 | 3.6 | 8.37 |  | USDA 2013 |
| Fruit | Cranberries, wild, bush, raw, Alaska, *Vibernum edule* | 7.9 | 40 | 1.4 |  | 3.36 | 3.6 | 8.37 |  | USDA 2013 |
| Fruit | Blackberries, wild, raw, Alaska, crowberry, *Rubus* spp. | 7.03 | 55.6 | 9 |  | 3.36 | 3.6 | 8.37 |  | USDA 2013 |
| Fruit | Blueberries, wild, raw, Alaska, *Vaccinium ovalifolium#* | 8.4 | 66.9 | 5.2 |  | 3.36 | 3.6 | 8.37 |  | USDA 2013 |
| Fruit | Blueberries, raw | 4.7 | 76.6 | 2.1 |  | 3.36 | 3.6 | 8.37 |  | USDA 2013 |
| Fruit | Rose Hips, wild | 3.9 | 34.2 | 0.8 |  | 3.36 | 3.6 | 8.37 |  | USDA 2013 |
| H. alpinum -- root | *Hedysarum alpinum* root§ | 12.6 | 33.8 | 1.5 |  | 3.47 | 4.07 | 8.37 |  | Coogan 2012 |
| Ungulate -- meat | Moose | 53 | 0 | 43 |  | 4.27 | 0 | 9.02 |  | Mattson et al. 2004 |
| Ungulate -- meat | Deer | 45.1 | 0 | 52.9 |  | 4.27 | 0 | 9.02 |  | Pritchard and Robbins 1990 |
| Ungulate -- meat | Deer, raw, game meat | 86.9 | 0 | 9.2 |  | 4.27 | 0 | 9.02 |  | USDA 2013 |
| Ungulate -- meat | Elk | 45 | 0 | 53 |  | 4.27 | 0 | 9.02 |  | Mattson et al. 2004 |
| Ungulate -- meat | Elk, game meat, raw | 89.6 | 0 | 5.7 |  | 4.27 | 0 | 9.02 |  | USDA 2013 |
| Ungulate -- meat | Moose meat, raw | 90.1 | 0 | 3 |  | 4.27 | 0 | 9.02 |  | USDA 2013 |
| Ungulate -- meat | Moose meat, raw, Alaska | 88.5 | 0 | 5.6 |  | 4.27 | 0 | 9.02 |  | USDA 2013 |
| Ungulate -- meat | Caribou, meat, raw† | 79.3 | 0 | 11.8 |  | 4.27 | 0 | 9.02 |  | USDA 2013 |
| Ungulate -- liver | Caribou, liver, raw, Alaska† | 52.4 | 23.8 | 13.6 |  | 4.27 | 4.11 | 9.02 |  | USDA 2013 |
| Ungulate -- liver | Moose, liver, braised, Alaska | 71.7 | 10.1 | 14.4 |  | 4.27 | 4.11 | 9.02 |  | USDA 2013 |
| Ungulate -- meat | Caribou, hind quarter, meat, raw, Alaska† | 82.6 | 0 | 12.3 |  | 4.27 | 0 | 9.02 |  | USDA 2013 |
| Ungulate -- liver | Lamb, liver, raw* | 71.2 | 6.2 | 17.5 |  | 4.27 | 4.11 | 9.02 |  | USDA 2013 |
| Ungulate -- liver | Beef, liver, raw* | 69.7 | 13.3 | 12.4 |  | 4.27 | 4.11 | 9.02 |  | USDA 2013 |
| Ungulate -- brain | Beef, brain, raw* | 45.8 | 4.4 | 43.4 |  | 4.27 | 4.11 | 9.02 |  | USDA 2013 |
| Ungulate -- brain | Lamb, brain, raw* | 50 | 0 | 41.25 |  | 4.27 | 4.11 | 9.02 |  | USDA 2013 |
| Ungulate -- brain | Veal, brain, raw* | 51 | 0 | 40.6 |  | 4.27 | 4.11 | 9.02 |  | USDA 2013 |
| Ungulate -- kidney | Beef, kidneys, raw* | 78.7 | 1.3 | 1.5 |  | 4.27 | 4.11 | 9.02 |  | USDA 2013 |
| Ungulate -- tongue | Caribou, tongue, raw†, Alaska | 36.5 | 0 | 60.8 |  | 4.27 | 4.11 | 9.02 |  | USDA 2013 |
| Ungulate -- tongue | Lamb, tongue, raw* | 46.2 | 0 | 52.1 |  | 4.27 | 4.11 | 9.02 |  | USDA 2013 |
| Ungulate -- tongue | Veal, tongue, raw* | 67.5 | 7.5 | 21.5 |  | 4.27 | 4.11 | 9.02 |  | USDA 2013 |
| Ungulate -- tongue | Beef, tongue, raw* | 42 | 10.4 | 45.7 |  | 4.27 | 4.11 | 9.02 |  | USDA 2013 |
| Ungulate -- marrow | Caribou, bone marrow, raw† | 8 | 0 | 91.1 |  | 4.27 | 0 | 9.02 |  | USDA 2013 |
| Ungulate -- eye | Caribou,eye, raw, Alaska† | 25.2 | 0 | 73.2 |  | 4.27 | 0 | 9.02 |  | USDA 2013 |
| Small mammal -- meat | Squirrel, raw, game meat | 81.7 | 0 | 3.21 |  | 4.27 | 0 | 9.02 |  | USDA 2013 |
| Small mammal -- meat | Squirrel, ground, meat, Alaska | 78.5 | 0 | 15.4 |  | 4.27 | 0 | 9.02 |  | USDA 2013 |
| Small mammal -- meat | Rabbit, wild, raw, game meat | 85.5 | 0 | 9.1 |  | 4.27 | 0 | 9.02 |  | USDA 2013 |

§Seasonal average nutritional values.

†Caribou are available to grizzly bears in the Kakwa ecosystem, but not the Yellowhead ecosystem.

*Ungulate proxy values from domestic animals.

‡Proxy for cow parsnip (Kunlein 1990).

±Minor food item.

**Proxy for green vegetation.

#This species of *Vaccinium* is found just outside the study area, but was included to represent *Vaccinum* spp.

**References**

Coogan SCP (2012) Getting to the root of the matter: grizzly bears and alpine sweetvetch in west-central Alberta, Canada. Masters thesis, Department of Renewable Resources, University of Alberta, Edmonton, AB, Canada. Available at: http://hdl.handle.net/10402/era.24865

Heuze V, Tran G, Hassoun P, Lebas F (2011) White clover (*Trifolium repens*), aerial part, fresh. Feedipedia.org. A programme by INRA, CIRAD, AFZ and FAO. Available: http://www.feedipedia.org/node/245. Accessed 03 December 2013.

Kuhnlein, HV (1990) Nutrient values in indigenous wild plant greens and roots used by the Nuxalk people of Bella Coola, British Columbia. J Food Compost Anal 3: 38-46.

Mattson DJ, Barber K, Maw R, Renkin R (2004) Coefficients of productivity for Yellowstone’s grizzly bear habitat. US Geological Survey, Biological Science Report USGS/BRD/BSR-2002-0007:1-76.

Merrill AL, Watt BK (1973) Energy value of foods: basis and derivation. Washington, DC: Agriculture Handbook 74, U.S. Government Printing Office.

Noyce KV, Kannowski PB, Riggs MR (1997) Black bears as ant-eaters: seasonal associations between bear myrmecophagy and ant ecology in north-central Minnesota. Can J Zool 75: 1671-1686.

Pritchard GT, Robbins CT (1990) Digestive and metabolic efficiencies of grizzly and black bears. Can J Zool 68: 1645-1651.

Rode KD, Robbins CT, Shipley LA (2001) Constraints on herbivory by grizzly bears. Oecologia 128: 62-71.

Swenson JE, Jansson A, Riig R, Sandegren F (1999) Bears and ants: myrmecophagy by brown bears in central Scandinavia. Can J Zool 77: 551-561.

Thomas VG, Prevett JP (1982) The role of horsetails (Equisetaceae) in the nutrition of northern-breeding geese. Oecologia 53: 359-363.

Tran G (2013) Timothy grass (Phleum pretense). Feedipedia.org. A programme by INRA, CIRAD, AFZ and FAO. Available: http://www.feedipedia.org/node/16886. Accessed 03 December 2013.

U. S. Department of Agriculture, Agricultural Research Service (2013) USDA National Nutrient Database for Standard Reference, Release 26. Available at: http://ndb.nal.usda.gov/. Accessed 03 December 2013.

Table 2 Metabolizable energy (kcal/g) contributions from individual macronutrients (crude protein, available carbohydrate, and lipid) in food items, sum of metabolizable energy (kcal/g) from all macronutrients, and metabolizable energy from individual macronutrients expressed as a percentage of the sum. Metabolizable energy was estimated using nutritional estimates and Atwater factors (Merrill and Watt 1973) given in Supporting Information S1 Table 1.

| Food Group | Diet Items |  | Protein | Carbohydrate | Lipid |  | Sum metabolizable energy |  | Protein | Carbohydrate | Lipid |
| --- | --- | --- | --- | --- | --- | --- | --- | --- | --- | --- | --- |
|  |  |  | (metabolizable energy (kcal/g)) | | |  | (kcal/g) |  | (% metabolizable energy) | | |
| ***Pre-green up*** |  |  |  |  |  |  |  |  |  |  |  |
| *H. alpinum* -- root | *Hedysarum alpinum* root |  | 0.44 | 1.38 | 0.13 |  | 1.94 |  | 23 | 71 | 6 |
| Ungulate -- meat | Moose |  | 3.46 | 0 | 1.35 |  | 4.81 |  | 72 | 0 | 28 |
| Ungulate -- meat | Moose meat, raw |  | 3.85 | 0 | 0.27 |  | 4.12 |  | 93 | 0 | 7 |
| Ungulate -- meat | Moose meat, raw, Alaska |  | 3.78 | 0 | 0.51 |  | 4.28 |  | 88 | 0 | 12 |
| Ungulate -- meat | Deer |  | 1.93 | 0 | 4.77 |  | 6.70 |  | 29 | 0 | 71 |
| Ungulate -- meat | Deer, raw, game meat |  | 3.71 | 0 | 0.83 |  | 4.54 |  | 82 | 0 | 18 |
| Ungulate -- meat | Elk |  | 3.42 | 0 | 1.62 |  | 5.04 |  | 68 | 0 | 32 |
| Ungulate -- meat | Elk, game meat, raw |  | 3.83 | 0 | 0.51 |  | 4.34 |  | 88 | 0 | 12 |
| Ungulate -- meat | Caribou, meat, raw |  | 3.39 | 0 | 1.06 |  | 4.45 |  | 76 | 0 | 24 |
| Ungulate -- meat | Caribou, hind quarter, meat, raw, Alaska |  | 3.53 | 0 | 1.11 |  | 4.64 |  | 76 | 0 | 24 |
| Ungulate -- liver | Caribou, liver, raw, Alaska |  | 2.24 | 0.98 | 1.23 |  | 4.44 |  | 50 | 22 | 28 |
| Ungulate -- liver | Moose, liver, braised, Alaska |  | 3.06 | 0.42 | 1.30 |  | 4.78 |  | 64 | 9 | 27 |
| Ungulate -- liver | Lamb, liver, raw |  | 3.04 | 0.25 | 1.58 |  | 4.87 |  | 62 | 5 | 32 |
| Ungulate -- liver | Beef, liver, raw |  | 2.98 | 0.55 | 1.12 |  | 4.64 |  | 64 | 12 | 24 |
| Ungulate -- brain | Beef, brain, raw |  | 1.96 | 0.18 | 3.91 |  | 6.05 |  | 32 | 3 | 65 |
| Ungulate -- brain | Lamb, brain, raw |  | 2.14 | 0 | 3.72 |  | 5.86 |  | 36 | 0 | 64 |
| Ungulate -- brain | Veal, brain, raw |  | 2.18 | 0 | 3.66 |  | 5.84 |  | 37 | 0 | 63 |
| Ungulate -- kidney | Beef, kidneys, raw |  | 3.36 | 0.05 | 0.14 |  | 3.55 |  | 95 | 2 | 4 |
| Ungulate -- tongue | Caribou, tongue, raw, Alaska |  | 1.56 | 0 | 5.48 |  | 7.04 |  | 22 | 0 | 78 |
| Ungulate -- tongue | Beef, tongue, raw |  | 1.79 | 0.43 | 4.12 |  | 6.34 |  | 28 | 7 | 65 |
| Ungulate -- tongue | Lamb, tongue, raw |  | 1.97 | 0 | 4.70 |  | 6.67 |  | 30 | 0 | 70 |
| Ungulate -- tongue | Veal, tongue, raw |  | 2.88 | 0.31 | 1.94 |  | 5.13 |  | 56 | 6 | 38 |
| Ungulate -- marrow | Caribou, bone marrow, raw |  | 0.34 | 0 | 8.22 |  | 8.56 |  | 4 | 0 | 96 |
| Ungulate -- eye | Caribou,eye, raw, Alaska |  | 1.08 | 0 | 6.60 |  | 7.68 |  | 14 | 0 | 86 |
| Small mammal -- meat | Squirrel, raw, game meat |  | 3.49 | 0 | 0.29 |  | 3.78 |  | 92 | 0 | 8 |
| Small mammal -- meat | Squirrel, ground, meat, Alaska |  | 3.35 | 0 | 1.39 |  | 4.74 |  | 71 | 0 | 29 |
| Small mammal -- meat | Rabbit, wild, raw, game meat |  | 3.65 | 0 | 0.82 |  | 4.47 |  | 82 | 0 | 18 |
| ***Graminoid & forb season*** | |  |  |  |  |  |  |  |  |  |  |
| Green vegetation | Celery |  | 0.37 | 1.07 | 0.31 |  | 1.75 |  | 21 | 61 | 18 |
| Green vegetation | Clover |  | 0.67 | 0.65 | 0.23 |  | 1.55 |  | 44 | 42 | 15 |
| Green vegetation | Graminoids |  | 0.50 | 0.60 | 0.18 |  | 1.29 |  | 39 | 47 | 14 |
| Green vegetation | Horsetails |  | 0.34 | 0.55 | 0.45 |  | 1.34 |  | 25 | 41 | 34 |
| Green vegetation | Dandelion |  | 0.73 | 1.41 | 0.41 |  | 2.55 |  | 29 | 55 | 16 |
| Green vegetation | Fireweed, young leaves, raw, Alaska |  | 0.57 | 0.08 | 0.53 |  | 1.18 |  | 48 | 7 | 45 |
| Green vegetation | Spinach |  | 0.81 | 0.59 | 0.38 |  | 1.78 |  | 46 | 33 | 21 |
| Green vegetation | Lettuce, green leaf, raw |  | 0.66 | 1.12 | 0.25 |  | 2.03 |  | 33 | 55 | 12 |
| Green vegetation | Cattail, Narrow Leaf, shoots, Typha lattifolia |  | 0.39 | 0.31 | 0.00 |  | 0.70 |  | 56 | 44 | 0 |
| Insects -- ants | Ant - Formica spp. Worker |  | 1.15 | 0.79 | 2.16 |  | 4.10 |  | 28 | 19 | 53 |
| Insects -- ants | Ant - Acanthomyops spp. workers 1 |  | 1.61 | 0.46 | 3.06 |  | 5.12 |  | 31 | 9 | 60 |
| Insects -- ants | Ant - Acanthomyops spp. workers 2 |  | 1.37 | 0.79 | 2.54 |  | 4.70 |  | 29 | 17 | 54 |
| Insects -- ants | Ant - Acanthomyops spp. pupae 1 |  | 1.87 | 0.60 | 3.22 |  | 5.70 |  | 33 | 11 | 57 |
| Insects -- ants | Ant - Acanthomyops spp. pupae 2 |  | 1.55 | 0.87 | 3.02 |  | 5.44 |  | 29 | 16 | 56 |
| Insects -- ants | Ant & pupae - *Camponotus herculeanus* |  | 1.87 | 0.91 | 1.75 |  | 4.53 |  | 41 | 20 | 39 |
| Insects -- ants | Ant & pupae - *Formica* spp. |  | 2.39 | 0.40 | 1.02 |  | 3.81 |  | 63 | 11 | 27 |
| Ungulate -- meat | Moose |  | 3.46 | 0 | 1.35 |  | 4.81 |  | 72 | 0 | 28 |
| Ungulate -- meat | Moose meat, raw |  | 3.85 | 0 | 0.27 |  | 4.12 |  | 93 | 0 | 7 |
| Ungulate -- meat | Moose meat, raw, Alaska |  | 3.78 | 0 | 0.51 |  | 4.28 |  | 88 | 0 | 12 |
| Ungulate -- meat | Deer |  | 1.93 | 0 | 4.77 |  | 6.70 |  | 29 | 0 | 71 |
| Ungulate -- meat | Deer, raw, game meat |  | 3.71 | 0 | 0.83 |  | 4.54 |  | 82 | 0 | 18 |
| Ungulate -- meat | Elk |  | 3.42 | 0 | 1.62 |  | 5.04 |  | 68 | 0 | 32 |
| Ungulate -- meat | Elk, game meat, raw |  | 3.83 | 0 | 0.51 |  | 4.34 |  | 88 | 0 | 12 |
| Ungulate -- meat | Caribou, meat, raw |  | 3.39 | 0 | 1.06 |  | 4.45 |  | 76 | 0 | 24 |
| Ungulate -- meat | Caribou, hind quarter, meat, raw, Alaska |  | 3.53 | 0 | 1.11 |  | 4.64 |  | 76 | 0 | 24 |
| Ungulate -- liver | Caribou, liver, raw, Alaska |  | 2.24 | 0.98 | 1.23 |  | 4.44 |  | 50 | 22 | 28 |
| Ungulate -- liver | Moose, liver, braised, Alaska |  | 3.06 | 0.42 | 1.30 |  | 4.78 |  | 64 | 9 | 27 |
| Ungulate -- liver | Lamb, liver, raw |  | 3.04 | 0.25 | 1.58 |  | 4.87 |  | 62 | 5 | 32 |
| Ungulate -- liver | Beef, liver, raw |  | 2.98 | 0.55 | 1.12 |  | 4.64 |  | 64 | 12 | 24 |
| Ungulate -- brain | Beef, brain, raw |  | 1.96 | 0.18 | 3.91 |  | 6.05 |  | 32 | 3 | 65 |
| Ungulate -- brain | Lamb, brain, raw |  | 2.14 | 0 | 3.72 |  | 5.86 |  | 36 | 0 | 64 |
| Ungulate -- brain | Veal, brain, raw |  | 2.18 | 0 | 3.66 |  | 5.84 |  | 37 | 0 | 63 |
| Ungulate -- kidney | Beef, kidneys, raw |  | 3.36 | 0.05 | 0.14 |  | 3.55 |  | 95 | 2 | 4 |
| Ungulate -- tongue | Caribou, tongue, raw, Alaska |  | 1.56 | 0 | 5.48 |  | 7.04 |  | 22 | 0 | 78 |
| Ungulate -- tongue | Beef, tongue, raw |  | 1.79 | 0.43 | 4.12 |  | 6.34 |  | 28 | 7 | 65 |
| Ungulate -- tongue | Lamb, tongue, raw |  | 1.97 | 0 | 4.70 |  | 6.67 |  | 30 | 0 | 70 |
| Ungulate -- tongue | Veal, tongue, raw |  | 2.88 | 0.31 | 1.94 |  | 5.13 |  | 56 | 6 | 38 |
| Ungulate -- marrow | Caribou, bone marrow, raw |  | 0.34 | 0 | 8.22 |  | 8.56 |  | 4 | 0 | 96 |
| Ungulate -- eye | Caribou,eye, raw, Alaska |  | 1.08 | 0 | 6.60 |  | 7.68 |  | 14 | 0 | 86 |
| Small mammal -- meat | Squirrel, raw, game meat |  | 3.49 | 0 | 0.29 |  | 3.78 |  | 92 | 0 | 8 |
| Small mammal -- meat | Squirrel, ground, meat, Alaska |  | 3.35 | 0 | 1.39 |  | 4.74 |  | 71 | 0 | 29 |
| Small mammal -- meat | Rabbit, wild, raw, game meat |  | 3.65 | 0 | 0.82 |  | 4.47 |  | 82 | 0 | 18 |
| H. alpinum -- root | *Hedysarum alpinum* root |  | 0.44 | 1.38 | 0.13 |  | 1.94 |  | 23 | 71 | 6 |
| ***Berry season*** |  |  |  |  |  |  |  |  |  |  |  |
| Fruit | *Vaccinium myrtilloides* |  | 0.16 | 2.34 | 0.24 |  | 2.74 |  | 6 | 85 | 9 |
| Fruit | *Vaccinium vits-idaea* |  | 0.15 | 2.40 | 0.28 |  | 2.82 |  | 5 | 85 | 10 |
| Fruit | *Vaccinum scoparium* |  | 0.16 | 2.35 | 0.37 |  | 2.89 |  | 6 | 82 | 13 |
| Fruit | Buffaloberry (*Shepherdia canadensis*) |  | 0.13 | 1.83 | 0.69 |  | 2.65 |  | 5 | 69 | 26 |
| Fruit | Crowberry (*Empetrum nigrum*) |  | 0.12 | 1.53 | 0.44 |  | 2.09 |  | 6 | 73 | 21 |
| Fruit | Raspberry, raw |  | 0.28 | 1.37 | 0.39 |  | 2.04 |  | 14 | 67 | 19 |
| Fruit | Raspberries, wild |  | 0.24 | 1.47 | 0.15 |  | 1.86 |  | 13 | 79 | 8 |
| Fruit | Strawberries, raw |  | 0.25 | 2.26 | 0.28 |  | 2.79 |  | 9 | 81 | 10 |
| Fruit | Gooseberries, raw, *Ribes* spp. |  | 0.25 | 1.75 | 0.40 |  | 2.39 |  | 10 | 73 | 17 |
| Fruit | Cranberries, wild, bush, raw, Alaska, *Vibernum edule* |  | 0.27 | 1.44 | 0.12 |  | 1.82 |  | 15 | 79 | 6 |
| Fruit | Blackberries, wild, raw, Alaska, crowberry, Rubus spp. |  | 0.24 | 2.00 | 0.75 |  | 2.99 |  | 8 | 67 | 25 |
| Fruit | Blueberries, wild, raw, Alaska, *Vaccinium ovalifolium* |  | 0.28 | 2.41 | 0.44 |  | 3.13 |  | 9 | 77 | 14 |
| Fruit | Blueberries, raw |  | 0.16 | 2.76 | 0.18 |  | 3.09 |  | 5 | 89 | 6 |
| Fruit | Rose Hips, wild |  | 0.13 | 1.23 | 0.07 |  | 1.43 |  | 9 | 86 | 5 |
| Green vegetation | Celery |  | 0.37 | 1.07 | 0.31 |  | 1.75 |  | 21 | 61 | 18 |
| Green vegetation | Clover |  | 0.48 | 0.64 | 0.23 |  | 1.34 |  | 35 | 48 | 17 |
| Green vegetation | Graminoids |  | 0.37 | 0.63 | 0.18 |  | 1.18 |  | 31 | 53 | 16 |
| Green vegetation | Horsetails |  | 0.27 | 0.62 | 0.34 |  | 1.24 |  | 22 | 50 | 28 |
| Green vegetation | Dandelion |  | 0.46 | 1.41 | 0.41 |  | 2.28 |  | 20 | 62 | 18 |
| Green vegetation | Fireweed, young leaves, raw, Alaska |  | 0.57 | 0.08 | 0.53 |  | 1.18 |  | 48 | 7 | 45 |
| Green vegetation | Spinach |  | 0.81 | 0.59 | 0.38 |  | 1.78 |  | 46 | 33 | 21 |
| Green vegetation | Lettuce, green leaf, raw |  | 0.66 | 1.12 | 0.25 |  | 2.03 |  | 33 | 55 | 12 |
| Green vegetation | Cattail, Narrow Leaf, shoots, Typha lattifolia |  | 0.39 | 0.31 | 0.00 |  | 0.70 |  | 56 | 44 | 0 |
| Insects -- ants | Ant - Formica spp. Worker |  | 1.15 | 0.79 | 2.16 |  | 4.10 |  | 28 | 19 | 53 |
| Insects -- ants | Ant - Acanthomyops spp. workers 1 |  | 1.61 | 0.46 | 3.06 |  | 5.12 |  | 31 | 9 | 60 |
| Insects -- ants | Ant - Acanthomyops spp. workers 2 |  | 1.37 | 0.79 | 2.54 |  | 4.70 |  | 29 | 17 | 54 |
| Insects -- ants | Ant - Acanthomyops spp. pupae 1 |  | 1.87 | 0.60 | 3.22 |  | 5.70 |  | 33 | 11 | 57 |
| Insects -- ants | Ant - Acanthomyops spp. pupae 2 |  | 1.55 | 0.87 | 3.02 |  | 5.44 |  | 29 | 16 | 56 |
| Insects -- ants | Ant & pupae - Camponotus herculeanus |  | 1.87 | 0.91 | 1.75 |  | 4.53 |  | 41 | 20 | 39 |
| Insects -- ants | Ant & pupae - Formica spp. |  | 2.39 | 0.40 | 1.02 |  | 3.81 |  | 63 | 11 | 27 |
| Ungulate -- meat | Moose |  | 2.86 | 0 | 2.62 |  | 5.48 |  | 52 | 0 | 48 |
| Ungulate -- meat | Moose meat, raw |  | 3.85 | 0 | 0.27 |  | 4.12 |  | 93 | 0 | 7 |
| Ungulate -- meat | Moose meat, raw, Alaska |  | 3.78 | 0 | 0.51 |  | 4.28 |  | 88 | 0 | 12 |
| Ungulate -- meat | Deer |  | 1.93 | 0 | 4.77 |  | 6.70 |  | 29 | 0 | 71 |
| Ungulate -- meat | Deer, raw, game meat |  | 3.71 | 0 | 0.83 |  | 4.54 |  | 82 | 0 | 18 |
| Ungulate -- meat | Elk |  | 2.65 | 0 | 3.25 |  | 5.89 |  | 45 | 0 | 55 |
| Ungulate -- meat | Elk, game meat, raw |  | 3.83 | 0 | 0.51 |  | 4.34 |  | 88 | 0 | 12 |
| Ungulate -- meat | Caribou, meat, raw |  | 3.39 | 0 | 1.06 |  | 4.45 |  | 76 | 0 | 24 |
| Ungulate -- meat | Caribou, hind quarter, meat, raw, Alaska |  | 3.53 | 0 | 1.11 |  | 4.64 |  | 76 | 0 | 24 |
| Ungulate -- liver | Caribou, liver, raw, Alaska |  | 2.24 | 0.98 | 1.23 |  | 4.44 |  | 50 | 22 | 28 |
| Ungulate -- liver | Moose, liver, braised, Alaska |  | 3.06 | 0.42 | 1.30 |  | 4.78 |  | 64 | 9 | 27 |
| Ungulate -- liver | Lamb, liver, raw |  | 3.04 | 0.25 | 1.58 |  | 4.87 |  | 62 | 5 | 32 |
| Ungulate -- liver | Beef, liver, raw |  | 2.98 | 0.55 | 1.12 |  | 4.64 |  | 64 | 12 | 24 |
| Ungulate -- brain | Beef, brain, raw |  | 1.96 | 0.18 | 3.91 |  | 6.05 |  | 32 | 3 | 65 |
| Ungulate -- brain | Lamb, brain, raw |  | 2.14 | 0 | 3.72 |  | 5.86 |  | 36 | 0 | 64 |
| Ungulate -- brain | Veal, brain, raw |  | 2.18 | 0 | 3.66 |  | 5.84 |  | 37 | 0 | 63 |
| Ungulate -- kidney | Beef, kidneys, raw |  | 3.36 | 0.05 | 0.14 |  | 3.55 |  | 95 | 2 | 4 |
| Ungulate -- tongue | Caribou, tongue, raw, Alaska |  | 1.56 | 0 | 5.48 |  | 7.04 |  | 22 | 0 | 78 |
| Ungulate -- tongue | Beef, tongue, raw |  | 1.79 | 0.43 | 4.12 |  | 6.34 |  | 28 | 7 | 65 |
| Ungulate -- tongue | Lamb, tongue, raw |  | 1.97 | 0 | 4.70 |  | 6.67 |  | 30 | 0 | 70 |
| Ungulate -- tongue | Veal, tongue, raw |  | 2.88 | 0.31 | 1.94 |  | 5.13 |  | 56 | 6 | 38 |
| Ungulate -- marrow | Caribou, bone marrow, raw |  | 0.34 | 0 | 8.22 |  | 8.56 |  | 4 | 0 | 96 |
| Ungulate -- eye | Caribou,eye, raw, Alaska |  | 1.08 | 0 | 6.60 |  | 7.68 |  | 14 | 0 | 86 |
| Small mammal -- meat | Squirrel, raw, game meat |  | 3.49 | 0 | 0.29 |  | 3.78 |  | 92 | 0 | 8 |
| Small mammal -- meat | Squirrel, ground, meat, Alaska |  | 3.35 | 0 | 1.39 |  | 4.74 |  | 71 | 0 | 29 |
| Small mammal -- meat | Rabbit, wild, raw, game meat |  | 3.65 | 0 | 0.82 |  | 4.47 |  | 82 | 0 | 18 |
| H. alpinum -- root | *Hedysarum alpinum* root |  | 0.44 | 1.38 | 0.13 |  | 1.94 |  | 23 | 71 | 6 |
| ***Late fall*** | |  |  |  |  |  |  |  |  |  |  |
| Fruit | *Vaccinium myrtilloides* |  | 0.16 | 2.34 | 0.24 |  | 2.74 |  | 6 | 85 | 9 |
| Fruit | *Vaccinium vits-idaea* |  | 0.15 | 2.40 | 0.28 |  | 2.82 |  | 5 | 85 | 10 |
| Fruit | *Vaccinum scoparium* |  | 0.16 | 2.35 | 0.37 |  | 2.89 |  | 6 | 82 | 13 |
| Fruit | Buffaloberry (*Shepherdia canadensis*) |  | 0.13 | 1.83 | 0.69 |  | 2.65 |  | 5 | 69 | 26 |
| Fruit | Crowberry (*Empetrum nigrum*) |  | 0.12 | 1.53 | 0.44 |  | 2.09 |  | 6 | 73 | 21 |
| Fruit | Raspberry, raw |  | 0.28 | 1.37 | 0.39 |  | 2.04 |  | 14 | 67 | 19 |
| Fruit | Raspberries, wild |  | 0.24 | 1.47 | 0.15 |  | 1.86 |  | 13 | 79 | 8 |
| Fruit | Strawberries, raw |  | 0.25 | 2.26 | 0.28 |  | 2.79 |  | 9 | 81 | 10 |
| Fruit | Gooseberries, raw, Ribes spp. |  | 0.25 | 1.75 | 0.40 |  | 2.39 |  | 10 | 73 | 17 |
| Fruit | Cranberries, wild, bush, raw, Alaska, *Vibernum edule* |  | 0.27 | 1.44 | 0.12 |  | 1.82 |  | 15 | 79 | 6 |
| Fruit | Blackberries, wild, raw, Alaska, crowberry, *Rubus* spp. | | 0.24 | 2.00 | 0.75 |  | 2.99 |  | 8 | 67 | 25 |
| Fruit | Blueberries, wild, raw, Alaska, *Vaccinium ovalifolium* |  | 0.28 | 2.41 | 0.44 |  | 3.13 |  | 9 | 77 | 14 |
| Fruit | Blueberries, raw |  | 0.16 | 2.76 | 0.18 |  | 3.09 |  | 5 | 89 | 6 |
| Fruit | Rose Hips, wild |  | 0.13 | 1.23 | 0.07 |  | 1.43 |  | 9 | 86 | 5 |
| H. alpinum -- root | *Hedysarum alpinum* root |  | 0.44 | 1.38 | 0.13 |  | 1.94 |  | 23 | 71 | 6 |
| Ungulate -- meat | Moose |  | 2.26 | 0 | 3.88 |  | 6.14 |  | 37 | 0 | 63 |
| Ungulate -- meat | Deer |  | 1.93 | 0 | 4.77 |  | 6.70 |  | 29 | 0 | 71 |
| Ungulate -- meat | Deer, raw, game meat |  | 3.71 | 0 | 0.83 |  | 4.54 |  | 82 | 0 | 18 |
| Ungulate -- meat | Elk |  | 1.92 | 0 | 4.78 |  | 6.70 |  | 29 | 0 | 71 |
| Ungulate -- meat | Elk, game meat, raw |  | 3.83 | 0 | 0.51 |  | 4.34 |  | 88 | 0 | 12 |
| Ungulate -- meat | Moose meat, raw |  | 3.85 | 0 | 0.27 |  | 4.12 |  | 93 | 0 | 7 |
| Ungulate -- meat | Moose meat, raw, Alaska |  | 3.78 | 0 | 0.51 |  | 4.28 |  | 88 | 0 | 12 |
| Ungulate -- meat | Caribou, meat, raw |  | 3.39 | 0 | 1.06 |  | 4.45 |  | 76 | 0 | 24 |
| Ungulate -- liver | Caribou, liver, raw, Alaska |  | 2.24 | 0.98 | 1.23 |  | 4.44 |  | 50 | 22 | 28 |
| Ungulate -- liver | Moose, liver, braised, Alaska |  | 3.06 | 0.42 | 1.30 |  | 4.78 |  | 64 | 9 | 27 |
| Ungulate -- meat | Caribou, hind quarter, meat, raw, Alaska |  | 3.53 | 0 | 1.11 |  | 4.64 |  | 76 | 0 | 24 |
| Ungulate -- liver | Lamb, liver, raw |  | 3.04 | 0.25 | 1.58 |  | 4.87 |  | 62 | 5 | 32 |
| Ungulate -- liver | Beef, liver, raw |  | 2.98 | 0.55 | 1.12 |  | 4.64 |  | 64 | 12 | 24 |
| Ungulate -- brain | Beef, brain, raw |  | 1.96 | 0.18 | 3.91 |  | 6.05 |  | 32 | 3 | 65 |
| Ungulate -- brain | Lamb, brain, raw |  | 2.14 | 0 | 3.72 |  | 5.86 |  | 36 | 0 | 64 |
| Ungulate -- brain | Veal, brain, raw |  | 2.18 | 0 | 3.66 |  | 5.84 |  | 37 | 0 | 63 |
| Ungulate -- kidney | Beef, kidneys, raw |  | 3.36 | 0.05 | 0.14 |  | 3.55 |  | 95 | 2 | 4 |
| Ungulate -- tongue | Caribou, tongue, raw, Alaska |  | 1.56 | 0 | 5.48 |  | 7.04 |  | 22 | 0 | 78 |
| Ungulate -- tongue | Lamb, tongue, raw |  | 1.97 | 0 | 4.70 |  | 6.67 |  | 30 | 0 | 70 |
| Ungulate -- tongue | Veal, tongue, raw |  | 2.88 | 0.31 | 1.94 |  | 5.13 |  | 56 | 6 | 38 |
| Ungulate -- tongue | Beef, tongue, raw |  | 1.79 | 0.43 | 4.12 |  | 6.34 |  | 28 | 7 | 65 |
| Ungulate -- marrow | Caribou, bone marrow, raw |  | 0.34 | 0 | 8.22 |  | 8.56 |  | 4 | 0 | 96 |
| Ungulate -- eye | Caribou,eye, raw, Alaska |  | 1.08 | 0 | 6.60 |  | 7.68 |  | 14 | 0 | 86 |
| Small mammal -- meat | Squirrel, raw, game meat |  | 3.49 | 0 | 0.29 |  | 3.78 |  | 92 | 0 | 8 |
| Small mammal -- meat | Squirrel, ground, meat, Alaska |  | 3.35 | 0 | 1.39 |  | 4.74 |  | 71 | 0 | 29 |
| Small mammal -- meat | Rabbit, wild, raw, game meat |  | 3.65 | 0 | 0.82 |  | 4.47 |  | 82 | 0 | 18 |

**References**

Merrill AL, Watt BK (1973) Energy value of foods: basis and derivation. Washington, DC: Agriculture Handbook 74, U.S. Government Printing Office.

**Estimation of non-selective ungulate consumption**

We created three models of non-selective ungulate consumption. For the first model, we assumed the proportion of edible ungulate parts given in Kuipers et al. (2010). These five components were: muscle; brain; liver; bone marrow; and adipose tissue. We then averaged the percent metabolizable energy macronutrient values in Table ESM2 for ungulate meat (muscle), brain, and liver. Bone marrow was not averaged since we had only a single estimate. For adipose tissue we used the value given for beef fat in Erlenbach et al. (2014). We then weighted metabolizable energy estimates for each ungulate component by the proportions given in Kuipers et al. (2010), and summed them. The formula for crude protein (% metabolizable energy) estimation is given below:

(0.902* Average Crude Protein Meat)) + (0.01* Average Crude Protein Brain) + (0.038* Average Crude Protein Liver) + (0.03*Crude Protein Bone Marrow) + (0.02*Crude Protein Adipose tissue)

This procedure was repeated for lipid and available carbohydrate and for each season.

**Table 3** Pregreen-up and Graminoid & Forb season non-selective ungulate consumption estimate using weighted proportions from Kuipers et al. (2010). Percent metabolizable energy was averaged from Table ESM2.

|  |  | Average % metabolizable energy | | |
| --- | --- | --- | --- | --- |
| Tissue | Proportion | Protein | Carbohydrate | Lipid |
| Meat | 0.902 | 75 | 0 | 25 |
| Brain | 0.01 | 35 | 1 | 64 |
| Liver | 0.038 | 60 | 12 | 28 |
| Bone marrow* | 0.03 | 4 | 0 | 96 |
| Adipose tissue* | 0.02 | 1 | 0 | 99 |
| Non-selective consumption | 1.00 | 70.1 | 0.5 | 29.4 |

* Single estimate (not averaged).

**Table 4** Berry season non-selective ungulate consumption estimate using weighted proportions from Kuipers et al. (2010). Percent metabolizable energy was averaged from Table ESM2.

|  |  | Average % metabolizable energy | | |
| --- | --- | --- | --- | --- |
| Tissue | Proportion | Protein | Carbohydrate | Lipid |
| Meat | 0.902 | 70 | 0 | 30 |
| Brain | 0.01 | 35 | 1 | 64 |
| Liver | 0.038 | 60 | 12 | 28 |
| Bone marrow* | 0.03 | 4 | 0 | 96 |
| Adipose tissue* | 0.02 | 1 | 0 | 99 |
| Non-selective consumption | 1.00 | 65.9 | 0.5 | 33.7 |

* Single estimate (not averaged).

**Table 5** Late fall non-selective ungulate consumption estimate using weighted proportions from Kuipers et al. (2010). Percent metabolizable energy was averaged from Table ESM2.

|  |  | Average % metabolizable energy | | |
| --- | --- | --- | --- | --- |
| Tissue | Proportion | Protein | Carbohydrate | Lipid |
| Meat | 0.902 | 66 | 0 | 34 |
| Brain | 0.01 | 35 | 1 | 64 |
| Liver | 0.038 | 60 | 12 | 28 |
| Bone marrow* | 0.03 | 4 | 0 | 96 |
| Adipose tissue* | 0.02 | 1 | 0 | 99 |
| Non-selective consumption | 1.00 | 62.7 | 0.5 | 36.8 |

* Single estimate (not averaged).

The second model of non-selective ungulate consumption for moose (minus hide and injesta) was derived from Hundertmark et al. (1997).Carbohydrate was not reported in Hundertmark et al. (1997). This estimate was used for all seasons.

Table 6 Percent metabolizable energy of individual macronutrients (crude protein, available carbohydrate, and lipid) in non-selectively consumed moose carcass derived from Hundertmark et al. (1997). Metabolizable energy was estimated using metabolic conversion (Atwater) factors from (Merrill and Watt 1973).

| Protein | Carbohydrate | Lipid |  | Protein | Carbohydrate | Lipid |  | Protein | Carbohydrate | Lipid |  | Sum  metabolizable energy |  | Protein | Carbohydrate | Lipid |
| --- | --- | --- | --- | --- | --- | --- | --- | --- | --- | --- | --- | --- | --- | --- | --- | --- |
| (% dry matter) | | |  | (metabolic conversion factors (kcal/g)) | | |  | (metabolizable energy (kcal/g)) | | |  | (kcal/g) |  | (% metabolizable energy) | | |
| 60.1 | 0 | 25.3 |  | 4.27 | 4.11 | 9.02 |  | 2.56627 | 0 | 2.28206 |  | 4.8483 |  | 53 | 0 | 47 |

The third model of non-selective ungulate consumption was derived from McCullough and Ullrey (1983) for white-tailed deer. We used data presented in Table 1 and Table 2 of McCullough and Ullrey (1983) to estimate the macronutrient composition of male and female deer of three different age classes (fawn, yearling and adult) minus hide (hair and skin), hooves, and antlers (where present). Estimates from McCullough and Ullrey (1983) were derived from fall and winter animals following a high acorn year and as such were in excellent body condition (high body fat). These estimates were used for all seasons, but may overestimate the fat content of ungulates during pregreen-up, graminoid and forb, and berry seasons.

Table 7 Percent metabolizable energy of individual macronutrients (crude protein, available carbohydrate, and lipid) in non-selectively consumed white-tailed deer carcass derived from McCullough and Ullrey (1983). Metabolizable energy was estimated using metabolic conversion (Atwater) factors from (Merrill and Watt 1973).

| Protein | Carbohydrate | Lipid |  | Protein | Carbohydrate | Lipid |  | Protein | Carbohydrate | Lipid |  | Sum  metabolizable energy |  | Protein | Carbohydrate | Lipid |
| --- | --- | --- | --- | --- | --- | --- | --- | --- | --- | --- | --- | --- | --- | --- | --- | --- |
| (% dry matter) | | |  | (metabolic conversion factors (kcal/g)) | | |  | (metabolizable energy (kcal/g)) | | |  | (kcal/g) |  | (% metabolizable energy) | | |
| Male -- fawn | | |  |  | | |  |  | | |  |  |  |  | | |
| 43.6 | 0 | 40.9 |  | 4.27 | 4.11 | 9.02 |  | 1.8604 | 0 | 3.6849 |  | 5.5453 |  | 34 | 0 | 66 |
| Male --yearling | | |  |  |  |  |  |  |  |  |  |  |  |  |  |  |
| 41.0 | 0 | 44.6 |  | 4.27 | 4.11 | 9.02 |  | 1.7512 | 0 | 4.0242 |  | 5.7754 |  | 30 | 0 | 70 |
| Male -- adult | | |  |  |  |  |  |  |  |  |  |  |  |  |  |  |
| 49.7 | 0 | 34.8 |  | 4.27 | 4.11 | 9.02 |  | 2.1219 | 0 | 3.1406 |  | 5.2625 |  | 40 | 0 | 60 |
| Female -- fawn | | |  |  |  |  |  |  |  |  |  |  |  |  |  |  |
| 46.3 | 0 | 37.6 |  | 4.27 | 4.11 | 9.02 |  | 1.9770 | 0 | 3.3922 |  | 5.3692 |  | 37 | 0 | 63 |
| Female -- yearling | | |  |  |  |  |  |  |  |  |  |  |  |  |  |  |
| 38.9 | 0 | 47.5 |  | 4.27 | 4.11 | 9.02 |  | 1.6621 | 0 | 4.2885 |  | 5.9506 |  | 28 | 0 | 72 |
| Female -- adult | | |  |  |  |  |  |  |  |  |  |  |  |  |  |  |
| 40.2 | 0 | 45.4 |  | 4.27 | 4.11 | 9.02 |  | 1.7172 | 0 | 4.0986 |  | 5.8159 |  | 30 | 0 | 70 |

**References**

Hundertmark KJ, Schwartz CC, Stephenson TA (1997) Estimation of body composition in moose. Federal Aid in Wildlife Restoration Study 1.42, Alaska Department of Fish and Game, Juneau, Alaska.

Kuipers RS, Luxwolda MF, Dijck-Brouwer DAJ, Eaton SB, Crawford MA, Cordain L, Muskiet FAJ (2010) Estimated macronutrient and fatty acid intakes from an East African paleolithic diet. Brit J Nutr 104:1666-1687 doi: 10.1017/S0007114510002679

McCullough DR, Ullrey DE (1983) Proximate mineral and gross energy composition of white-tailed deer. J Wildl Manag 47: 430-441.

Merrill AL, Watt BK (1973) Energy value of foods: basis and derivation. Washington, DC: Agriculture Handbook 74, U.S. Government Printing Office.

**Estimation of optimal ungulate composition**

In order to estimate the ratio of protein to lipid in an optimally balanced ungulate on a mass basis we simply back-calculated from the optimal ratio of protein to lipid (% metabolizable energy) using a spreadsheet. Carbohydrate was considered negligible for this extimate.

Table 8 Back-calculation of the ratio of protein to lipid on a mass basis in an optimally balanced non-selectively consumed ungulate carcass.

| Food Group | Ratio of  protein :lipid | Protein | Lipid | Protein | Lipid | Protein | Lipid | Sum metabolizable energy  (kcal/g) | Protein | Lipid |
| --- | --- | --- | --- | --- | --- | --- | --- | --- | --- | --- |
|  |  | (% dry matter) | | (metabolic conversion factors (kcal/g))* | | (metabolizable energy (kcal/g)) | |  | (% metabolizable energy) | |
| Whole Ungulate  balanced | 0.433 | 30.200 | 69.800 | 4.27 | 9.02 | 1.290 | 6.296 | 7.586 | 17.000 | 83.000 |
| Whole Ungulate  balanced | 0.433 | 21.633 | 50.000 | 4.27 | 9.02 | 0.924 | 4.510 | 5.434 | 17.000 | 83.000 |
| Whole Ungulate  balanced | 0.433 | 10.817 | 25.001 | 4.27 | 9.02 | 0.462 | 2.255 | 2.717 | 17.000 | 83.000 |

Conversely, the ratio of lipid to protein on a mass basis is 2.311:1.

**Limited analysis of monthly macronutrient estimates for vegetation**

We used previously unpublished nutritional estimates of green vegetation in order to aid in food modelling. These estimates were used to inform macronutrient estimates of grizzly bear plant foods, since published data was limiting (particularly for total dietary fibre). Methods follow Coogan (2012) as well as Coogan et al. (2012) for crude protein estimates. Note that lipid and available carbohydrate (by subtraction) were not estimated.

**Table 9** Monthly mean nutritional estimates (% dry matter), standard deviation (*SD*), and sample size (*n*) of herbaceous grizzly bear food samples collected in west-central Alberta, Canada. Instances where standard deviation could not be calculated due to n = 1 are denoted by (-).

| Food | Estimate | Month | | | | |
| --- | --- | --- | --- | --- | --- | --- |
|  |  | May | June | July | August | September |
|  |  | mean (*SD*) *n* | mean (*SD*) *n* | mean (*SD*) *n* | mean (*SD*) *n* | mean (*SD*) *n* |
| Cow parsnip (*Heracleum lanatum*) | Crude protein |  | 26.8 (-) 1 | 15.1 (2.4) 3 | 9.7 (2.0) 4 |  |
|  | Ash |  | 12.2 (-) 1 | 19.9 (2.0) 3 | 19.5 (3.8) 4 |  |
|  | Crude fibre |  | 26.6 (-) 1 | 32.1 (1.9) 3 | 30.3 (6.9) 4 |  |
|  | Total dietary fibre |  | 57.1 (-) 1 | 67.5 (2.6) 3 | 64.7 (10.3) 4 |  |
|  | Digestible protein |  | 20.2 (-) 1 | 9.8 (2.1) 3 | 5.1 (1.7) 4 |  |
|  | Digestible dry matter |  | 21.9 (-) 1 | 7.5 (3.6) 3 | 11.4 (14.4) 4 |  |
|  | Digestibly energy |  | 26.2 (-) 1 | 14.3 (3.0) 3 | 17.5 (11.9) 4 |  |
| Clover (*Trifolium* spp.) | Crude protein | 30.5 (-) 1 | 32.7 (5.6) 2 | 19.5 (3.5) 7 | 16.1 (1.5) 8 | 15.3 (0.9) 3 |
|  | Ash |  | 10.0 (-) 1 | 10.1 (1.2) 6 | 9.2 (1.3) 5 | 9.1 (2.3) 2 |
|  | Crude fibre | 14.7 (-) 1 | 13.2 (-) 1 | 26.5 (6.7) 5 | 29.8 (4.2) 4 | 29.0 (-) 1 |
|  | Total dietary fibre |  |  | 56.1 (10.1) 5 | 60.9 (6.8) 4 | 60.4 (-) 1 |
|  | Digestible protein | 23.4 (-) 1 | 25.4 (4.9) 2 | 13.7 (3.1) 7 | 10.7 (1.4) 8 | 10.0 (0.8) 3 |
|  | Digestible dry matter |  |  | 23.3 (14.1) 5 | 16.6 (9.4) 4 | 17.3 (-) 1 |
|  | Digestibly energy |  |  | 27.4 (11.7) 5 | 21.9 (7.8) 4 | 22.4 (-) 1 |
| Dandelion (*Taraxacum officinale*) | Crude protein | 29.8 (-) 1 | 19.9 (2.2) 4 | 16.1 (5.4) 2 | 9.7 (0.7) 3 |  |
|  | Ash |  | 15.4 (3.7) 4 | 16.4 (2.5) 2 |  |  |
|  | Crude fibre |  | 11.3 (0.8) 4 | 17.0 (1.3) 2 |  |  |
|  | Total dietary fibre |  | 34.9 (1.9) 4 | 43.6 (2.7) 2 |  |  |
|  | Digestible protein | 22.8 (-) 1 | 14.1 (2.0) 4 | 10.7 (4.7) 2 | 5.1 (0.6) 3 |  |
|  | Digestible dry matter |  | 52.8 (2.7) 4 | 40.7 (3.8) 2 |  |  |
|  | Digestibly energy |  | 51.9 (2.2) 4 | 41.8 (3.2) 2 |  |  |
| Horsetail (*Equisetum arvense*) | Crude protein | 26.8 (-) 1 | 29.6 (-) 1 | 21.9 (2.2) 2 | 17.1 (4.3) 3 |  |
|  | Ash | 17.8 (-) 1 | 15.3 (-) 1 | 20.3 (1.5) 2 |  |  |
|  | Crude fibre | 19.4 (-) 1 | 16.5 (-) 1 | 22.1 (-) 1 |  |  |
|  | Total dietary fibre | 47.6 (-) 1 |  | 52.4 (-) 1 |  |  |
|  | Digestible protein | 20.2 (-) 1 | 22.7 (-) 1 | 15.8 (2.0) 2 | 11.6 (3.8) 3 |  |
|  | Digestible dry matter | 35.1 (-) 1 |  | 28.5 (-) 1 |  |  |
|  | Digestibly energy | 37.2 (-) 1 |  | 31.7 (-) 1 |  |  |

Coogan SCP (2012) Getting to the root of the matter: grizzly bears and alpine sweetvetch in west-central Alberta, Canada. Masters thesis, Department of Renewable Resources, University of Alberta, Edmonton, AB, Canada. Available at: http://hdl.handle.net/10402/era.24865

Coogan SCP, Nielsen SE, Stenhouse GB (2012) Spatial and temporal heterogeneity creates a “brown tide” in root phenology and nutrition. ISRN Ecol 2012: 10. doi:10.5402/2012/618257

**Modelled nutrient estimates for green vegetation**

Since complete or suitable macronutrient estimates for some green vegetation were unknown to us, we combined some estimates from different sources to estimate available carbohydrate. Macronutrient estimates, and literature source, for modelled green vegetation estimates are as follows:

Clover (Graminoid and Forb Season)

100% - 27.6 % crude protein -2.7% lipid^†^ – 40.1% TDF – 11.5% ash^†^ = 18.1% available carbohydrate

*Source*: Rode et al 2001; ^†^Tran 2013

Clover (Berry Season)

100% - 19.5 % crude protein^†^ -2.7% lipid^†^ – 48.3% TDF – 11.5% ash^†^ = 8.4% available carbohydrate

*Source*: Rode et al 2001; ^†^Tran 2013

*Note*: we used the lowest value in the range given in Tran 2013 to estimate crude protein content, because we thought the value given in Rode et al. 2001 was too high to reflect protein decline with phenological advancement.

Graminoids (Graminoid and Forb Season)

100% - 20.5 % crude protein -2.2% lipid^‡^ – 52.4% TDF – 8.0% ash^‡^ = 16.9% available carbohydrate

*Source:* Rode et al. 2001; ^‡^Hueze et al 2011

Graminoids (Berry Season)

100% - 15.0 % crude protein* -2.2% lipid^‡^ – 57.1% TDF – 8.0% ash^‡^ = 17.7% available carbohydrate

*Source:* Rode et al 2001; *Mattson et al. 2004; ^‡^Hueze et al 2011

Horsetails (Graminoid and Forb Season)

100% - 14.0 % crude protein -5.4% lipid – 50.0% TDF^§^ – 15.3% ash = 15.3% available carbohydrate

*Source*: Thomas and Prevett 1982; ^§^Coogan 2012

Horsetails (Berry Season)

100% - 11.2 % crude protein -4.1% lipid – 50.0% TDF^§^ – 17.2% ash = 17.5% available carbohydrate

*Source*: Thomas and Prevett 1982; ^§^Coogan 2012

Dandelion (Graminoid and Forb Season)

We used the May crude protein estimate (29.8%) from Appendix Table ESM8, rather than the USDA value (18.8%), in order to better represent the high protein content of dandelion early in the year.

References for the above calculations are listed following Table ESM1.

**Nutritional analyses of buffaloberry**

After performing proximate nutritional analysis for buffaloberry fruit (Coogan 2012) we found that crude protein (N × 6.25) estimates for whole fruit (seed and pulp) were higher than expected.

**TABLE 10** Proximate nutritional estimates (mean percent dry mass, standard deviation (*SD*) and sample size (*n*)) for buffaloberry whole fruit.

|  | Crude protein  (%) | Crude fibre  (%) | Ash  (%) | Fat  (%) | Total dietary fibre (%) | Available carbohydrate (%) |
| --- | --- | --- | --- | --- | --- | --- |
|  | mean (*SD*) *n* | | | | | |
| *1. Shepherdia canadensis*† | **14.4** (3.6) 10 | **8.8** (1.7) 10 | **2.7** (0.7) 10 | **8.3** (1.1) 10 | **23.9** (2.7) 10 | **50.7** (6.5) 10 |
| *2. Shepherdia canadensis* | **19.8** (2.4) 8 | **10.1** (1.9) 8 | **3.0** (0.3) 8 |  | **26.3** (2.7) 8 |  |

† Indicates samples that were analyzed at an independent external laboratory.

We hypothesized that high nitrogen estimates may be due to the single large seed, and since buffaloberry seeds are typically found whole in bear scats, we estimated the relative contribution of nitrogen from the seed and pulp of russet buffaloberry fruit. We used only estimates for buffaloberry pulp in the RMT grizzly-bear-nutrient-space analysis

Calculations for the dry weight, mass of N, % N, and % crude protein content of pulp in buffaloberry fruit

We determined the dry mass of individual buffaloberry whole fruits and single seeds by drying samples at 110°C to a constant mass. We determined the crude protein content of individual whole fruit (*n*=19) and individual seeds (*n*=12) following Coogan (2012) and Coogan et al. (2012). We determined the crude protein content of pulp in individual fruit by subtraction.

**Table 11** Mean dry mass, percent nitrogen content (dry mass), mass of nitrogen, and estimated percent crude protein content* (%N × 6.25) of individual *Shepherdia canadensis* seeds, whole berries, and pulp. See below for calculations.

*Shepherdia canadensis* berry statistics *SD* *n*

Mean dry mass seed: 7.9 mg 1.4 12

Mean dry mass whole berry: 32.3 mg 7.3 19

Mean dry mass pulp†: 24.4 mg - -

Mean % N per seed: 11.1% 1.6 19

Mean % N whole berry: 3.2% 0.4 8

Mean % N pulp: 0.6% - -

Mean mass N per seed: 0.8737 mg - -

Mean mass N whole berry: 1.0229 mg - -

Mean mass N pulp†: 0.1492 mg - -

Mean % crude protein* per seed: 69.2% 9.9 19

Mean % crude protein* whole berry: 19.8% 2.4 8

Mean % crude protein* pulp: 3.8% - -

* Assuming nitrogen from protein.

† By subtraction.

Calculation used to estimate crude protein content in buffaloberry fruit pulp

1. Dry weight of pulp in Shepherdia berry:

Average dry weight whole berry – average dry weight seeds = estimated average dry weight pulp

0.0323 g – 0.0079 g = **0.0244 g**

2. Mass of Nitrogen in Shepherdia berry pulp:

1.) 7.9 mg/seed × 11.06% N = 0.8737 mg N/seed

2.) 32.3 mg/whole berry × 3.167% N = 1.0229 mg N/whole berry

3.) mg N in whole berry – mg N in seed = mg N in pulp

1.0229 mg N – 0.8737 mg N = **0.1492 mg N in pulp**

3.) % N in Shepherdia berry pulp:

(0.1492 mg N in pulp ÷ 24.4 mg pulp in berry) × 100% = **0.6115% N in pulp**

4.) % Crude protein in Shepherdia berry pulp:

0.6115% N in pulp × 6.25 = **3.8219% crude protein in pulp**

While the crude protein content of buffaloberry fruit pulp is similar to other fruits, the crude protein content of the seed is high. We suspect that high nitrogen levels in buffaloberry seed may be due to non-protein nitrogenous compounds rather than protein. We, therefore, suggest further research into the nature of nitrogen in buffaloberry fruit.

**References**

Coogan SCP (2012) Getting to the root of the matter: grizzly bears and alpine sweetvetch in west-central Alberta, Canada. Masters thesis, Department of Renewable Resources, University of Alberta, Edmonton, AB, Canada. Available at: http://hdl.handle.net/10402/era.24865

Coogan SCP, Nielsen SE, Stenhouse GB (2012) Spatial and temporal heterogeneity creates a “brown tide” in root phenology and nutrition. ISRN Ecol 2012: 10. doi:10.5402/2012/618257
